# Comprehensive Characterization of Stem Cell Landscape Identifies Novel Stemness-Relevant Genes for Nasopharyngeal Carcinoma Therapy

**DOI:** 10.3390/cancers18030422

**Published:** 2026-01-28

**Authors:** Dahua Xu, Bocen Chen, Yutong Shen, Guoqing Deng, Peihu Li, Jiale Cai, Jiayao Chen, Jing Bai, Yuyue Tian, Man Xiao, Hong Wang, Hongyan Jiang, Wangwei Cai, Bo Wang, Kongning Li

**Affiliations:** 1 School of Intelligent Medicine and Technology, Big Data Research Center, Hainan General Hospital and Hainan Affiliated Hospital, Hainan Medical University, Haikou 571199, China; xudahua1209@muhn.edu.cn (D.X.);; 2 Hainan Engineering Research Center for Health Big Data, Hainan Medical University, Haikou 571199, China; 3 Key Laboratory of Biochemistry and Molecular Biology, Hainan Medical University, Haikou 571000, China; chenbocen921017@163.com (B.C.);

**Keywords:** stemness, tumor immune microenvironment, drug response, prognosis, NPC

## Abstract

Background: Metastasis and recurrence account for the failure of nasopharyngeal carcinoma (NPC) treatment. Growing evidence indicates the dominant roles of cancer stem cells (CSCs) in tumor progression and therapy resistance. However, the heterogeneity of CSCs and potential stemness-related markers in NPC patients are still largely unknown. Methods: Consensus clustering was first applied to identify robust stemness subtypes for NPC patients based on the activities of stem cell gene sets. The differences in clinical outcomes, tumor immune microenvironment (TIME), and drug response were compared between subtypes. The stemness-related markers were prioritized via weighted gene correlation network analysis (WGCNA) and Cox regression, and verified through in vitro experiments. Results: NPC patients were classified into C1 and C2 subtypes. The C2 subtype exhibited higher activities of stem cell gene sets, worse prognosis, and aggressive tumor progression thus defined as stem cell-like tumor phenotype. The exclusionary relationships between tumor stemness and TIME infiltration were observed. The efficacy of several drugs and immunotherapy varied between NPC stemness subtypes. Through the WGCNA and survival analysis, we found that PSMC3IP, NABP2, CDC45, and HJURP were stemness-relevant genes. Sphere formation assays and analysis of the protein expression of stem cell markers by Western blotting revealed the roles of PSMC3IP, NABP2, CDC45, and HJURP in promoting CSC properties. Moreover, these genes were found to be related to the therapeutic effect of telomerase inhibitor in CCK8 experiments. Conclusions: This study systematically characterized two NPC subtypes with distinct stemness features, clinical outcomes, and TIME features. Novel stemness-related markers will provide valuable targets against metastatic or recurrent NPC.

## Simple Summary

Nasopharyngeal carcinoma (NPC) is a rare type of head and neck malignant tumor with specific geographical distribution. Growing evidence reveals the dominant roles of cancer stem cells (CSCs) in tumor progression and therapy resistance. However, the heterogeneity of CSCs and potential stemness-related markers for NPC patients are still largely unknown. We undertook a systematical analysis, dissecting the stemness heterogeneity of NPC patients. We classified NPC patients into two optimal clusters based on stemness-related gene lists, which were characterized by distinct clinical outcomes, immune phenotypes, and drug response. The immune cell exclusion is associated with the presence of the NPC stem cell-like phenotype. In particular, novel stemness-related markers including PSMC3IP, NABP2, CDC45, and HJURP were prioritized via WGCNA and Cox regression analysis. Moreover, their stemness traits in NPC cells were verified by Western blot, sphere formation, and CCK8 assays. Our results will provide valuable targets against metastatic or recurrent NPC.

## 1. Introduction

Nasopharyngeal carcinoma (NPC) is a rare type of head and neck malignant tumor with a specific geographical distribution: it is particularly prevalent in Southeast Asia, especially in southern China [1]. Due to the anatomic localization of nasopharynx, the combination of chemotherapy and radiotherapy has become the primary treatment and significantly improved the survival rate for NPC patients [2]. Nevertheless, approximately 10–20% of NPC patients fail to benefit from first-line chemotherapy and experience tumor progression [3]. Drug resistance, recurrence, tumor invasion, and metastasis are the main risk factors for NPC mortality. The molecular mechanisms and potential biomarkers related to risky NPC patients are still largely unknown.

Cancer stem cells (CSCs), a class of pluripotent cells, are characterized by self-renewal, proliferation without restriction, and multidirectional differentiation [4]. Evidence has shown that CSCs represent a subpopulation of tumor mixture that exhibit resistance to chemotherapy and the “stemness” of cancer cells is closely related to tumor progression, metastasis, and recurrence [5]. In addition, CSCs have been considered a driver of intratumoral heterogeneity [5]. Global negative associations between stemness and the tumor immune microenvironment (TIME) as well as anticancer immunity were observed across cancer types [6]. However, the characteristics of the stem cells and their impact on drug sensitivity and TIME components in NPC remains poorly understood.

The development of deep sequencing and computational methods has provided valuable stemness markers for cancer precision medicine. For instance, a one-class logistic regression machine learning algorithm (OCLR) was proposed to extract stemness features associated with the oncogenic state at both the transcriptomic (mRNAsi) and epigenetic levels (mDNAsi) [7]. Combining the utilization of mRNAsi and molecular experiments, Zheng et al. identified a novel stemness-related gene, COLEC12, that attenuates CSC traits in colorectal cancer [8]. NUSAP1 was found to interact with RACK1 to stimulate STAT3 nuclear translocation, thus enhancing hepatocellular carcinoma stemness. Moreover, elevated gene expression was associated with early HCC recurrence and poor survival, which indicates the value of NUSAP1 as a treatment target [9]. Thus, efforts to identify stemness-related markers could address the problems of metastasis and recurrence for NPC patients.

This study aimed to characterize the stem cell landscape and identify stemness-related markers for NPC patients. We classified NPC patients into two optimal clusters with distinct stemness features and clinical outcomes. The NPC stemness subtype excluded immune cell infiltration and further affected the chemotherapy and immunotherapy response. Hub genes associated with NPC stemness subtypes and prognosis were prioritized by WGCNA. Among them, PSMC3IP, NABP2, CDC45, and HJURP were verified by in vitro experiments to serve as novel NPC stemness markers. Conclusively, the essential genes obtained from stemness landscape analysis will improve the clinical strategies for NPC treatment.

## 2. Materials and Methods

### 2.1. NPC Datasets Collection and Preprocessing

The RNA sequencing data of 113 fresh, treatment-naïve nasopharyngeal carcinoma tumor samples was first collected from the GEO (Gene Expression Omnibus, https://www.ncbi.nlm.nih.gov/geo/, accessed on 24 October 2022) with accession ID GSE102349. The fragments per kilobase per million mapped reads (FPKM) values indicating gene expression level and data on clinical stage, progression-free survival (PFS), and the original tumor microenvironment-based subtypes were obtained from Zhang’s study [10]. The transcriptome of head and neck squamous cell carcinoma (HNSC) was obtained from The Cancer Genome Atlas (TCGA, https://portal.gdc.cancer.gov/, accessed on 3 December 2022) portal. Moreover, four microarrays of NPC and normal tissues were selected as external datasets (GSE12452, GSE13597, GSE53819, and GSE61218). The microarrays of NPC CSCs and non-CSCs determined by spheroid culture were collected from the GEO database with accession ID GSE36124. The gene expression level was quantified by the average signal of corresponding probes. Detailed information on the NPC datasets is provided in Table S1.

### 2.2. Stemness Estimation and NPC Stemness Subtype Identification

A total of 474 genes related to 29 stem cell gene sets were obtained from the Molecular Signature Database (MSigDB, v2022.1.Hs). The single-sample GSEA (ssGSEA) algorithm was used to estimate the 29 gene set activities in the NPC samples [11]. Then, the ConsensusClusterPlus package was applied to identify the NPC subtypes based on the stem cell gene lists [12]. Partitioning around medoids cluster was applied based on the Euclidean distance. The procedure was run with 1000 iterations and a subsampling ratio of 0.8 to ensure dependability.

To verify the availability of NPC stemness subtypes, we selected 26 stemness gene sets (Homo sapiens) from StemChecker, which determines stemness-related genes via transcriptome analysis, RNAi screens, transcription factor (TF) target gene sets, retrieval of the literature and computational derivation [13]. The ssGSEA method was applied to estimate the activities of StemChecker gene lists for NPC samples. We also computed the messenger RNA expression-based stemness index (mRNAsi), which reflects the stemness levels, based on the OCLR machine-learning algorithm [7]. The Wilcoxon rank-sum test was used to compare the differences in stemness features among NPC subtypes. All stemness-related gene sets we used are provided in Table S2.

### 2.3. Tumor Immune Microenvironment Infiltration, Antitumor Immunoactivity and Immune Signature Exploration

Seven computational frameworks with high confidence (CIBERSORT, CIBERSORT-ABS, QUANISEQ, TIMER, EPIC, MCPCOUNTER, and xCell) were used to estimate the components of the immune microenvironment for NPC samples via the TIMER 2.0 website (http://timer.cistrome.org/, accessed on 10 November 2022) [14]. Antitumor immunoactivity was measured according to a previous study by four features as follows [15]: (a) ESTIMATE score, which represents tumor purity [16]; (b) immune score obtained from xCell, which reflects integrative immune activity [17]; (c) MHC score, which was computed by the average gene expression of the MHC-I set [18]; and (d) CYT score, which was calculated as the geometric mean of the GZMA and PRF1 expression levels [19]. The immune signature gene lists, including immune checkpoints, human leukocyte antigen (HLA), tumor-infiltrating lymphocytes (TILs), macrophages/monocytes, lymphocyte infiltration, TGF-β response, IFN-γ response, and wound healing were collected from previous studies [20]. ssGSEA was used to estimate the activity of immune signatures. The Wilcoxon rank-sum test was used to compare the differences in the immune scores of the NPC subtypes mentioned above.

The gene sets for B-cell signature were collected from Arian and Alexnader studies [21,22]. B-cell signature scores were calculated by averaging the expression levels of related genes. We also downloaded raw CEL files of B cells and 19 other immune cell types from the GEO database for the Affymetrix HG-U133_Plus 2.0 platform according to a previous study [23]. All CEL files were uniformly preprocessed with background correction and quantile normalization by the RMA algorithm. The limma R package was used to identify specifically expressed genes in B cells with log2FC > 1 and FDR < 0.05 as thresholds.

### 2.4. Drug Sensitivity and Immunotherapy Response Prediction

The half-maximum inhibitory concentration (IC50) of chemotherapy drugs for NPC samples was predicted by oncoPredict [24], a ridge regression-based machine learning method, that used the drug treatment transcriptome of cancer cell lines in Genomics of Drug Sensitivity in Cancer (GDSC v2). In addition, the Tumor Immune Dysfunction and Exclusion (TIDE) algorithm was used to predict the immunotherapy response of NPC patients [25]. Patients with high TIDE scores have a higher probability of tumor immune evasion and are thus less likely to benefit from immunotherapy. The dysfunction and exclusion scores indicated the T-cell dysfunction and exclusion potential of the tumor, respectively.

### 2.5. Weighted Gene Correlation Network Analysis (WGCNA)

WGCNA was applied to construct a coexpression network of NPC patients based on the median absolute deviation (MAD) of the top 10,000 genes [26]. The coexpression similarity matrix Sij was first built by the Pearson correlation coefficient r between any two genes (genei and genej). Then, a weighted adjacency matrix aij was constructed by choosing the soft thresholding power β = 3. The aij was further transformed into a topological overlap matrix (TOM) and correlative dissimilarity matrix (1-TOM), and highly interconnected gene modules were identified with a module minimum size of 30. The associations between module eigengene (ME), which reflected the first principal component of network modules, and stemness subtypes, stemness features, and immune-related scores were estimated based on Pearson correlation. The hub genes of specific modules were determined by gene significance (GS) > 0.4 and module membership (MM) > 0.6. Functional enrichment analysis of hub genes was performed by the Metascape portal [27].

### 2.6. Survival Analysis

Univariate Cox regression was performed to filter PFS-related hub genes. The median expression of hub genes was used as the cutoff to divide NPC patients into two groups, and the log-rank test was applied to compare PFS between groups. Only genes with both hazard ratio (HR) *p* < 0.05 and log-rank *p* < 0.05 were retained. We further estimated the prognosis of hub genes related to disease free survival (DFS, which is related to tumor recurrence) for HNSC patients in the TCGA cohort via GEPIA2 tools [28].

### 2.7. Cell Culture

Normal human nasopharyngeal epithelial cells (NP69) were purchased from Shanghai Jin Yuan Biotechnology. NPC cell lines including HNE1, CNE2, 5-8F, and SUNE-1 were purchased from Xiamen Yimei Cell Bank. Cells were cultured at 37 °C with 5% CO_2_ in high-glucose Dulbecco’s modified Eagle’s medium (DMEM) containing 10% fetal serum (Gibco-Life Technologies, Carlsbad, CA, USA) and 1% penicillin and streptomycin.

### 2.8. RT-qPCR

Total RNA was extracted from the cells via the EastepSuper Total RNA Extraction Kit (LS1040, Promega, Shanghai, China). Total RNA was reverse transcribed into cDNA using the SuperMix for qPCR (gDNA digester plus) (H7101160, YEASEN, Shanghai, China) Reverse Transcription Kit. Then, qPCR Green Master Mix (H6103020, YEASEN, Shanghai, China) was used to detect the relative expression of each gene according to the instructions. The primer sequences for each gene are shown in Table S3. The expression levels of the target genes were normalized to those of GAPDH, and the results were reported as fold changes relative to the threshold cycle (CT value).

### 2.9. siRNA Transfection

Cells were plated in 6-well plates, and the medium was replaced with serum- and double antibody-free medium. The GND reagent (Gidina Biologics) was mixed (10 μg/μL) before complex preparation and transfection; 100 μL of medium was added to 1.5 mL sterile centrifuge tubes followed by 4 μL of siRNA 1 μg (Shanghai, Abbott Laboratories). The siRNA sequences are shown in Table S4. Meanwhile, 80 μL of the medium was added to a separate 1.5 mL sterile tube, followed by 20 μL of GND reagent (10 μg/μL). The medium was added to the siRNA mixture and allowed to stand at room temperature for 15 min before immediate transfection. Finally, we added 200 μL of the transfection mixture to the wells at a concentration of 200 μg/mL of GND reagent and shaken the plate gently to distribute the mixture. The cells were incubated at 37 °C and after 6 h the medium was changed to a complete medium.

### 2.10. Western Blotting

Total protein was extracted from cells. Western blotting was performed by SDS-PAGE gels and transferred onto polyvinylidene fluoride (PVDF) membranes. After blocking with 5% nonfat milk, the membranes were separately probed with primary antibodies. Our primary antibodies are listed below: PSMC3IP rabbit pAb (#100560, 1:500, Thermo Fisher, Waltham, MA, USA), NABP2 rabbit pAb (#100654, 1:1000, Thermo Fisher, Massachusetts, USA), CDC45 rabbit pAb (#3673, 1:1000, CST, Boston, MA, USA), HJURPR rabbit mAb (#80508, 1:1000, CST, Boston, USA), LGR5 rabbit pAb (#75850, 1:1000, Abcam, MA, USA), CD44 rabbit pAb (#189524, 1:1000, Abcam, USA), SOX2 rabbit pAb (#113131, 1:1000, Absin, Shanghai, China), NANOG rabbit pAb (#109250, 1:1000, Abcam, MA, USA), OCT4 rabbit pAb (#19857, 1:1000, Abcam, MA, USA), and GAPDH rabbit mAb (#2118S, 1:1000, CST, Boston, USA). After incubation at 4 °C overnight, the membranes were washed and incubated with the appropriate secondary antibody (1:1000, Beyotime, Shanghai, China). The blotted proteins were detected using ECL (Thermo Fisher, Massachusetts, USA) reagents.

### 2.11. Cell Sphere Formation Assay

Cell digestion and counting was performed on ultralow adsorption cell culture plates (Corning^®^ Costar^®^ ultralow adsorption porous plates). Approximately 1000 cells were added to each well of a 6-well plate for cell cultivation and incubated for approximately 10 days. After incubation, the cells were observed under a microscope to determine their sphericity, and sphere formation was calculated using the following formula: sphere formation = (number of spheres/number of cells plated) × 100.

### 2.12. CCK8 Assay

Cell viability was detected by the CCK8 assay according to the manufacturer’s instructions (CK04, Dojindo, Kyushu Island, Japan). Cells were seeded into a 96-well culture plate at a density of 2 × 103 cells/well. After 48 h, 10 μL of CCK8 solution was added to each well and incubated for 1 h at 37 °C. Then, the viability was recorded based on the optical density (OD) value detected at 450 nm.

### 2.13. Statistical Analysis

Statistical analysis was performed using R software (4.4.1) for data processing and modeling. Wilcoxon rank-sum test was applied to estimate the differences between two groups. The correlation between two vectors was estimated by Pearson correlation analysis. Experimental data were analyzed with GraphPad Prism software, version 8.0. In all experimental figures, error bars represent standard deviation (SD), and *p*-values were determined using one-way ANOVA or *t*-tests. A *p* value > 0.05 was considered nonsignificant (n.s.); * *p* < 0.05; ** *p* < 0.01; *** *p* < 0.001.

## 3. Results

### 3.1. Stemness Subtypes Reflected Different Clinical Outcomes of NPC

**Figure 1:**
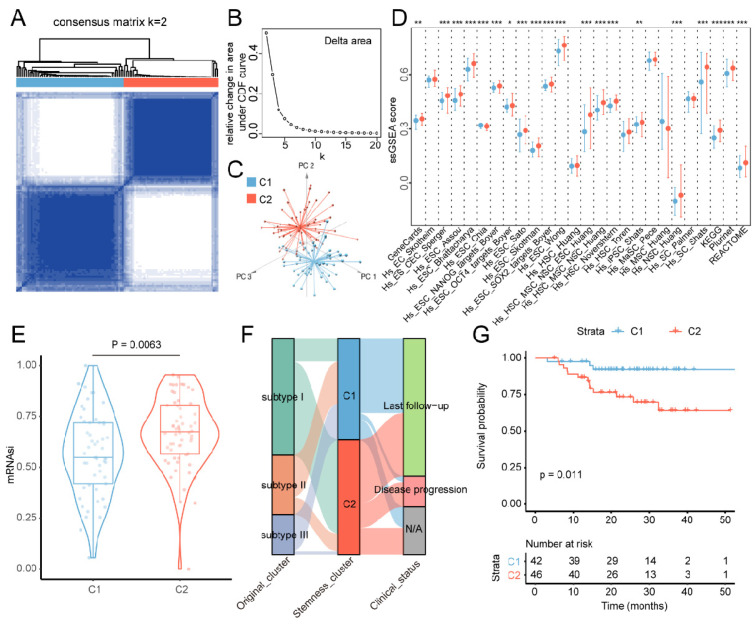
Characteristics of stem cell landscape in NPC patients. (**A**) Consensus clustering matrix of NPC cancer samples for k = 2 via MsigDB stemness features. (**B**) Relative change in area under CDF curve for k = 2 to k = 20. (**C**) PCA dimensionality reduction plot of NPC patients. Individual samples are colored by the subtypes. (**D**) Box plots showing the activities of StemChecker stemness categories estimated by ssGSEA in NPC subtypes. (**E**) The difference in mRNAsi score between NPC clusters. (**F**) Alluvial diagram of distributions for NPC patients with original molecular cluster, stemness cluster, and clinical outcomes. (**G**) The Kaplan–Meier plot of NPC cohort. Patients were divided into C1 and C2 subtypes, and *p* value was estimated via the log rank test. * *p* < 0.05; ** *p* < 0.01; *** *p* < 0.001.

Stem cell landscape analysis facilitates the understanding of the carcinogenic mechanism and therapeutic strategies for multiple cancer types [6]. To investigate the stemness heterogeneity of NPC samples, we first performed consensus clustering based on the activities of manually curated stem cell gene sets. The NPC patients were grouped into 2 optimal clusters (C1 and C2) with a stable distribution based on the relative change in the area under the CDF curve (Figure 1A,B). We also performed principal component analysis (PCA) to visualize the clusters with stem cell features, where C1 and C2 were separated into three dimensions (Figure 1C). We next enrolled 55 human stemness-related gene lists to verify the robustness of the NPC stemness subtypes. Approximately 70% of stemness gene lists exhibited increased activities in the C2 cluster (20/29 and 19/26 in MSigDB and StemChecker, respectively, Figure S1 and Figure 1D). Moreover, the mRNAsi scores of C2 patients were significantly higher than those of C1 patients (*p* < 0.05, Figure 1E). After evaluating the three stemness features mentioned above, we summarized that the C2 cluster was attributed to a stem cell-like tumor phenotype.

Since the NPC patients were originally clustered into three subtypes with distinct clinical outcomes based on genome-wide mRNA expression profiles (GSE102349) [10], we next estimated the relationship between the new stemness NPC subtypes and the original clusters. We found that a large proportion of patients with subtype I, which had the worst prognosis, was assigned to the C2 cluster, while the C1 cluster was mainly formed by subtype II and III patients (Figure 1F). Regarding PFS, we found that C2 patients had a worse prognosis than C1 patients (log-rank *p* < 0.05, Figure 1G). Moreover, the prognostic capacity of the stemness subtype was better than that of the original subtype (*p* = 0.011 vs. *p* = 0.08). In addition, the ratio of late-stage patients was elevated in the C2 cluster, whereas all stage I patients were assigned to the C1 cluster (Chi-square test, *p* = 0.0577, Figure S2A). These results were consistent with observations that cancer stem cells are the key drivers in aggressive stages of tumor progression [29,30,31,32]. Stemness heterogeneity existed in the NPC patients and could reflect the difference in clinical outcomes.

### 3.2. NPC Stemness Subtype Excluded Immune Cell Infiltration

**Figure 2:**
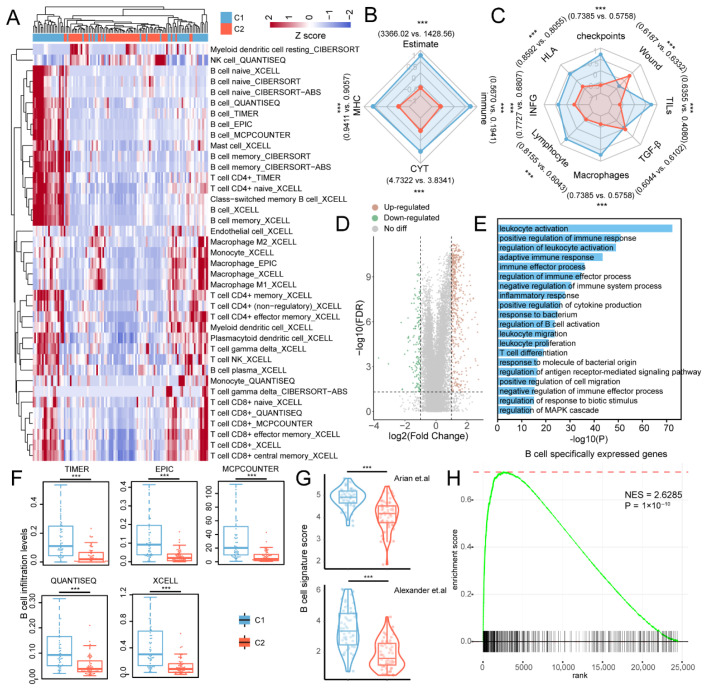
TIME heterogeneity in NPC subtypes. (**A**) Heatmap showing the immune cell infiltrations obtained from CIBERSORT, CIBERSORT-ABS, QUANISEQ, TIMER, EPIC, MCPCOUNTER, and xCell for NPC subtypes. The rows represent estimated immune cell infiltrations and columns represent NPC patients. (**B**,**C**) The mean value of scaled antitumor immunoactivity and immune signature scores between NPC subtypes. Characters on the outside of radar map represent the mean value of unscaled immune scores (C1 vs. C2). (**D**) Volcano plot showing the DE genes between NPC subtypes. (**E**) Functional enrichment analysis of DE genes. (**F**) The B cell infiltration levels between NPC subtypes for multiple computational algorithms. (**G**) The B cell signature scores between NPC subtypes [21,22]. (**H**) GSEA analysis of B cell specifically expressed genes between C1 and C2 clusters. *** *p* < 0.001.

Previous studies have reported negative associations between stemness and immune cell infiltration for most cancers [6]. We next investigated the extent of TIME heterogeneity in NPC subtypes. First, the proportion of intratumoral TILs quantified by histopathologic examination was higher in C1 cluster, indicating the potential inverse correlation between stemness and tumor immune cell infiltration (Figure S2B). To determine the differences in the infiltration levels of specific cell types, we integrated seven well-known computational frameworks to capture the immune cell component of NPC patients. In general, individual stemness subtypes varied significantly in terms of the relative proportion in the TIME; for most immune cells, infiltration levels were increased in the C1 cluster, and only the infiltrating levels of resting myeloid dendritic cell (CIBERSORT) and NK cells (QUANTISEQ) were elevated in the C2 cluster (|LogFC| > 1, FDR < 0.05, Figure 2A). Next, we considered the ESTIMATE, CYT, MHC and immune scores to estimate antitumor immunoactivity. All four scores were significantly higher in the C1 cluster, indicating that C1 patients exhibited lower tumor purity, greater tumor-killing activity, stronger antigen presentation capacity and higher immunoactivity (Figure 2B). In addition, the C1 cluster also exhibited higher activities of most immune signatures (Figure 2C), while no difference was observed for the TGF-β response. In particular, we found that the stemness subtype (C2 cluster) showed increased activity of the wound healing signature, which is consistent with the contributions of stem cells to wound healing and tumor formation [33]. Differentially expressed gene (DEG) analysis was performed and identified 635 dysregulated genes in NPC subtypes, including 503 upregulated and 132 downregulated genes in the C1 cluster compared to the C2 cluster (|LogFC| > 1, FDR < 0.05, Figure 2D, Table S5). The top three upregulated genes were GP2, LTF, and IGHE, and the top three downregulated genes were SOX10, DKK1, and ELSPBP1. LTF has been reported as a tumor suppressor gene that inhibits the NPC cell growth [34]. Conversely, the high expression of SOX10 and DKK1 indicates poor survival in NPC and HNSC patients [35,36]. These results were consistent with the clinical outcomes of C1 and C2 patients. Furthermore, the DEGs were significantly enriched in immune-related biological processes such as leukocyte activation, positive regulation of immune response, leukocyte migration and proliferation (Figure 2E).

The formation of tertiary lymphoid structures benefited from the increased B lymphocytes in the TIME, which prolonged the PFS of NPC patients [37,38]. We next explored the relationship between B cells and NPC stemness and found that regulation of B-cell activation was significantly enriched in the DEGs of NPC subtypes (Figure 2E). Moreover, robust elevation of B-cell lineage infiltration in the C1 cluster was observed across multiple TIME estimation methods (Figure 2A,F), indicating negative associations between stemness and B cells infiltration. We also manually collected two cohorts of B-cell signatures and estimated their activities in NPC patients. We found that the B-cell signature scores were all increased in the C1 cluster (Figure 2G). Considering the rank order based on the fold change in protein-coding genes, the B-cell specifically expressed genes were significantly upregulated in the C1 cluster compared to the C2 cluster (Figure 2H). These results suggest that stemness inversely associated with immune cell infiltration, especially for the B–lineage cells, in NPC patients.

### 3.3. Drug Sensitivity and Immunotherapy Response of the NPC Stemness Subtype

**Figure 3:**
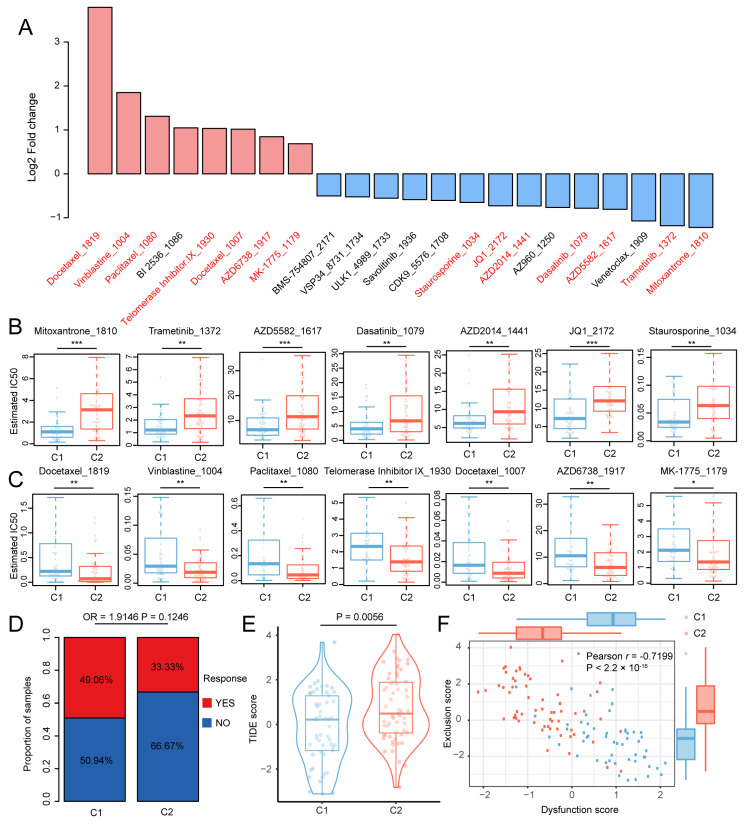
Cancer stemness affected the drug response for NPC patients. (**A**) The differences in estimated IC50 values for chemotherapeutics between NPC subtypes. Only the IC50 values of drugs exhibited significantly varied between NPC clusters were shown. (**B**,**C**) The estimated IC50 values between NPC subtypes for drugs which reported to be associated with NPC treatment. (**D**) Bar plot showing the proportions of patients who responded to immunotherapy in NPC subtypes. (**E**) Box plot showing the differences in TIDE score between NPC subtypes. (**F**) The negative correlation between exclusion and dysfunction score for NPC patients. * *p* < 0.05; ** *p* < 0.01; *** *p* < 0.001.

Cancer stem cells have been reported to be involved in chemoresistance, which impedes the treatment of recurrent NPC patients [39]. To screen potential chemotherapeutic drugs related to NPC stemness subtypes, we estimated the IC50 values of drugs provided by GDSC v2 for each NPC sample and retained drugs with an average IC50 less than 20. As shown in Figure 3A, the IC50s of 22 drugs significantly varied between NPC subtypes (|Log2FC| > 0.5, FDR < 0.05). The IC50s of eight drugs were increased in the C1 cluster, while 14 drugs showed the opposite trend in the C2 cluster. Notably, we identified 13 drugs that had been reported to be associated with NPC treatment (Table S6). Among them, the estimated IC50 values of mitoxantrone, trametinib, AZD5582, dasatinib, AZD2014, JQ1 and staurosporine were significantly lower in the C1 cluster, implying that patients in the C1 cluster might be more sensitive to these drugs (Figure 3B). Meanwhile, the C2 cluster was more sensitive to docetaxel, vinblastine, paclitaxel, telomerase inhibitor IX, AZD6738 and MK-1775 (Figure 3C). Interestingly, evidence has shown the suppressive ability of Pin2 telomeric repeat factor 1-interacting telomerase inhibitor 1 (PinX1) in NPC stemness, indicating the potential therapeutic function of telomerase inhibitor IX in NPC treatment [40].

The intricate TIME and stemness status have a non-negligible impact on immunotherapy responses for cancers [41]. We next estimated the immunotherapy responses of the NPC subtypes through the TIDE algorithm. Consistent with the immune ‘Hot’ phenotype with a higher infiltration of immune cells and immune signature, patients in the C1 cluster benefited more from immunotherapy treatment than those in the C2 cluster (49.06% vs. 33.33, OR = 1.9146, Figure 3D). Moreover, the TIDE score and T-cell exclusion score in the C2 cluster were significantly increased, while the T-cell dysfunction score was decreased (Figure 3E,F). Consistent with the inverse correlations between T-cell dysfunction and T-cell exclusion across multiple cancer types [25], a strong negative relationship was also observed for these two features in NPC samples (Figure 3F). These results highlight the roles of stemness in chemotherapy and immunotherapy resistance for NPC patients.

### 3.4. Identification of Stemness-Associated Module via WGCNA

**Figure 4:**
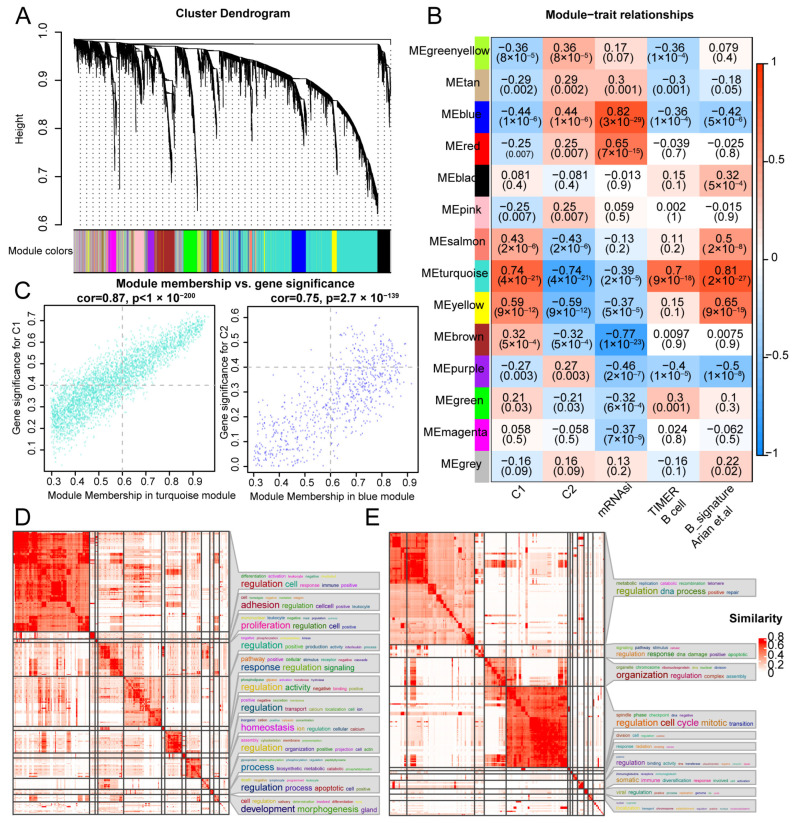
Identification of hub genes by WGCNA for NPC subtypes. (**A**) The cluster dendrogram developed by the weighted correlation coefficients; each color represents a co-expression gene module. (**B**) Heatmap shows correlation between the gene modules and clinical traits, which include C1, C2, mRNAsi, B cell infiltration, and B cell signature. (**C**) Scatter plot displaying relationship of module membership (MM) in turquoise and blue module with gene significance for NPC clusters. (**D**,**E**) Function enrichment of hub genes from turquoise and blue modules. GO terms were clustered into groups based on gene similarities.

As NPC subtypes carry distinct stemness and immune features, they affect the clinical outcomes and drug treatment response. We further selected the top 10,000 genes with expression deviations in the NPC samples to perform WGCNA. The thresholding power β was set as 3 (scale-free R2  =  0.86) to build a scale-free network (Figure S3). Then, the 14 network modules were identified via the clustering dendrogram to ensure that the genes with similar expression patterns clustered (Figure 4A). After combining clinical traits, we filtered the turquoise module that showed the most positive correlation with the C1 cluster, B-cell infiltration, and signature but was negatively related to C2 and stemness signature (Figure 4B). Conversely, the blue module was found to be most positively associated with the C2 cluster and NPC stemness (Figure 4B). According to the threshold of GS > 0.4 and MM > 0.6, a total of 1341 and 141 intersecting candidate hub genes were obtained from the turquoise and blue modules, respectively, for further analysis (Figure 4C).

To explore the potential biological functions of the hub genes in the turquoise and blue modules, GO enrichment analyses were performed via the Metascape portal. Consistent with the immune-inflamed characteristics of the C1 cluster, hub genes in the turquoise module were significantly enriched in immune regulation processes including leukocyte activation, regulation of cell activation, and regulation of B-cell activation (Figure 4D and Figure S4A). Moreover, the enriched GO terms for the hub genes from the blue module were associated with cell cycle and metabolism processes such as the DNA metabolic process, regulation of cell cycle process, and chromosome organization (Figure 4E and Figure S4B). The results suggest that NPC subtype-related modules play distinct roles in tumor progression.

### 3.5. Priority of Prognostic Hub Genes for the NPC Stemness Module

**Figure 5:**
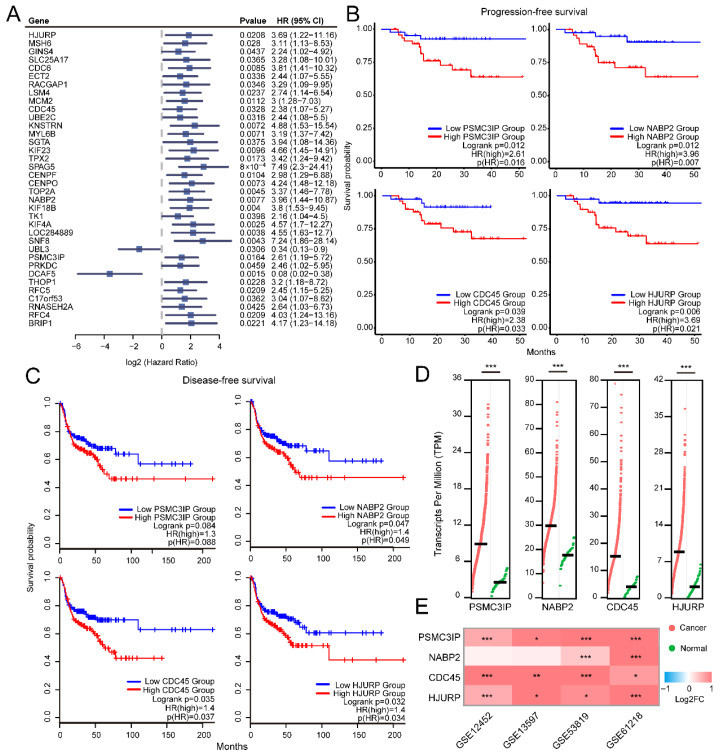
Stemness module hub genes were related to NPC survival. (**A**) Univariate Cox regression results for the hub genes from blue module. (**B**) Kaplan–Meier curves of progression-free survival for patients with high or low PSMC3IP, NABP2, CDC45, and HJURP expression in NPC dataset. (**C**) Kaplan–Meier curves of disease-free survival for patients with high or low PSMC3IP, NABP2, CDC45, and HJURP expression in TCGA HNSC dataset. (**D**) Expression level of PSMC3IP, NABP2, CDC45, and HJURP in TCGA HNSC tumor and adjacent normal samples. (**E**) Expression change in PSMC3IP, NABP2, CDC45, and HJURP in NPC tissues compared to controls in multiple datasets. * *p* < 0.05; ** *p* < 0.01; *** *p* < 0.001.

Due to the highest correlation between the blue module and NPC stemness features, we next focused on the hub genes from this module to prioritize prognostic biomarkers. Univariate Cox regression analysis was first applied to estimate the relationship between each hub gene and NPC PFS. In total, we identified 35 protein-coding genes and a long-noncoding RNA LOC284889 that were significantly associated with NPC survival (hazard ratio *p* < 0.05 and log-rank *p* < 0.05, Figure 5A). Among them, UBL3 and DCAF5 were found to serve as protective factors for NPC PFS, while the others were risk factors in patients. Furthermore, the DFS of the TCGA HNSC cohort was used to verify the robustness of the prognostic ability of the hub genes. We found that the high expression of PSMC3IP, NABP2, CDC45, and HJURP was associated with worse survival in both the NPC PFS and HNSC DFS datasets, implying the associations of these genes with disease progression and recurrence (Figure 5B,C). The expression patterns of these genes were next explored, and we found that the expression level was significantly increased in NPC stemness-like cluster and HNSC tumor samples (Figure S5 and Figure 5D). The increased expression of these four genes in tumor tissue was observed across multiple external NPC datasets (Figure 5E). Moreover, elevated trends of PSMC3IP, CDC45, and HJURP were observed in NPC CSCs compared to non-CSCs (Figure S6). Altogether, the stemness-related hub genes were strongly related to tumor survival and processes.

### 3.6. PSMC3IP, NABP2, CDC45, and HJURP Facilitate CSC Traits of NPC Cells

**Figure 6:**
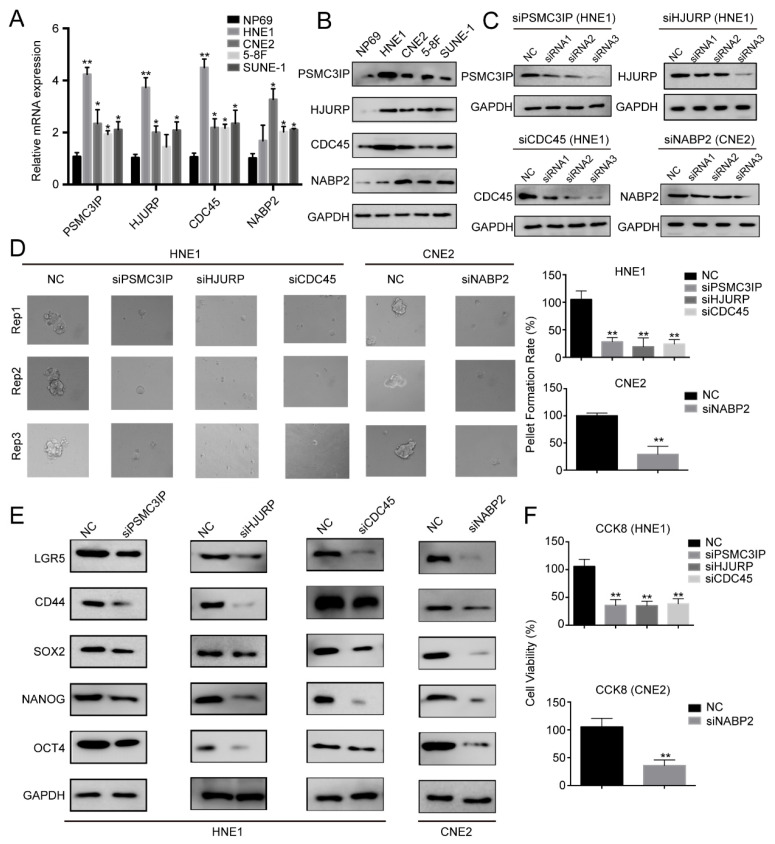
Verified stemness traits of PSMC3IP, HJURP, CDC45, and NABP2 in NPC cells. (**A**,**B**) The mRNA and protein levels of PSMC3IP, HJURP, CDC45, and NABP2 in normal nasopharyngeal epithelial and NPC cell lines. (**C**) The protein levels of PSMC3IP, HJURP, CDC45 in HNE1 and NABP2 in CNE2 cells transduced with siRNA. (**D**) Representative images of HNE1 and CNE2 cell spheres after transfection with NC and siRNAs. Bar plots show the pellet formation rate of NPC cells. (**E**) Western blot showed the expression of stemness-related markers LGR5, CD44, SOX2, NANOG and OCT4 after transfection with NC and siRNAs. (**F**) CCK-8 assays show that the inhibition of PSMC3IP, HJURP, CDC45 in HNE1 and NABP2 in CNE2 cells decreased cell proliferation. * represent *p* < 0.05, ** represent *p* < 0.01. The uncropped blots and molecular weight markers are shown in Supplementary Figure S10.

The capacities of hub genes as NPC stemness markers were next validated via in vitro experiments. The expression levels of PSMC3IP, NABP2, CDC45, and HJURP were first detected in four NPC cell lines (HNE1, CNE2, 5-8F, and SUNE-1) and a normal nasopharyngeal epithelial cell line (NP69). We found that the expression levels of these genes were all increased in NPC cell lines compared to normal nasal cells at both the RNA and protein levels (Figure 6A,B). Among them, PSMC3IP, HJURP, and CDC45 were highly expressed in HNE1 cells, and NABP2 was highly expressed in CNE2 cells. Thus, siRNA technology was applied to knock down these genes in the corresponding cell lines with the highest expression. The specific siRNAs successfully inhibited PSMC3IP, NABP2, and CDC45 expression in HNE1 cells, as well as the expression of NABP2 in CNE2 cells (Figure 6C and Figure S7). We further performed sphere formation assays and Western blot assays to investigate the effect of PSMC3IP, NABP2, CDC45, and HJURP on the stemness of NPC cells. Sphere formation assays indicated that the sphere numbers and sizes of NPC cells were markedly attenuated after the knockdown of PSMC3IP, NABP2, CDC45, and HJURP (Figure 6D). Moreover, the knockdown of these hub genes inhibited the protein levels of several stem cell markers, including LGR5, CD44, SOX2, NANOG and OCT4 (Figure 6E). No changes in CD44 and SOX2 were observed after the interference of CDC45 and NABP2, respectively. In addition, CCK-8 analysis indicated that the proliferation of NPC cells was delayed after the silencing of hub genes (Figure 6F). To investigate the relationships between hub genes and NPC treatment, we randomly selected the telomerase inhibitor, which is suitable for stemness-like NPC subtype patients, to perform further analysis. The CCK-8 assay indicated that the telomerase inhibitor effectively inhibited the proliferation potential of HNE1 and CNE2 cells (IC50: 6 μM, Figure S8A). However, the proliferation of NPC cells was not changed between the inhibition of hub gene groups and the combination of siRNA and drug treatment groups (Figure S8B–E). These observations were consistent with our predicted results that NPC patients with stemness features (C2 cluster with upregulated PSMC3IP, NABP2, CDC45, and HJURP) benefited from telomerase inhibitor treatment, while the drug was not suitable for C1 patients with downregulated PSMC3IP, NABP2, CDC45, and HJURP. Taken together, these results revealed the stemness traits of PSMC3IP, NABP2, CDC45, and HJURP in NPC cells.

## 4. Discussion

CSCs are responsible for cancer growth and progression and have become novel targets for NPC therapy [42]. The heterogeneity of stem cell status and the dynamic equilibrium of CSCs and other cell populations within tumor microenvironments bring challenges to the application of precision treatment [43]. In this study, we characterized the stem cell landscape of NPC patients and identified NPC subtypes with distinct stemness features estimated from multicenter datasets, including MSigDB, StemChecker, and mRNAsi. We found that the precise clustering of NPC patients based on stemness activities improved the clinical outcomes compared to the original molecular classification strategy. Thus, our NPC stemness-related subtype classification system could be worthwhile in guiding personalized precision therapy.

Exploring the intricate interactions between stemness and TIME will aid in understanding CSC-mediated immune regulation and the development of stem cell therapeutic strategies [44]. In accordance with the fact that stemness contributes to the immunosuppressive TIME of cancers, we found that the NPC stem cell-like phenotype exhibited lower infiltration levels for multiple immune cell types, especially for B cell, CD4 T-cell and CD8 T-cell lineages. We further considered antitumor immunoactivity and immune signatures and found that almost all immune features were inhibited in the NPC stemness subtype. For instance, a lower IFN gamma score was observed in the C2 cluster, which implies that C2 was unable to suppress tumor growth [45]. Moreover, the patients in the C1 cluster, who had high immune scores and were less likely to have a stemness phenotype, were sensitive to immunotherapy. We found that the C1 cluster was mainly derived from the original subtype II and III patients, while the C2 cluster was mainly formed by subtype I patients. Patients with disease progression were overlapped with patients in the C2 cluster and original subtype I, which consisted with the worse prognosis of the C2 cluster. The immune landscape of the original NPC categories was explored, and the infiltration of most immune cell types in subtype I was significantly decreased compared with that in the other subtypes, which was consistent with the worse survival status of subtype I patients (Figure S9A). Moreover, the infiltration level of B-cell lineages was elevated in subtype II, while T-cell lineages were enriched in subtype III. These results may partially reveal the immune heterogeneity and different clinical outcomes of NPC subtypes. We also found that a large proportion of patients with subtype II benefited from immunotherapy (Figure S9B). According to the estimates obtained from the TIDE algorithm, the patients in our subtypes exhibited distinct distributions of TIDE score, T-cell exclusion score, and T-cell dysfunction score, while no significant differences between original subtype I and III were observed, which may be another superiority of stemness-based classification for NPC patients. All these results revealed the influence of stemness on immune cell exclusion and immunotherapy effects in NPC patients.

We next assessed the sensitivity to chemotherapy in different NPC subtypes to seek potential therapeutic medicine for risky patients. There were 22 and 31 targeted drugs for our NPC subtypes and original subtypes, respectively (Figure 3A and Figure S9C). Among them, 15 drugs were identified as candidates for both NPC classification types. In addition, drugs including vinblastine, paclitaxel, BI 2536, BMS-754807, savolitinib, CDK9_5576, and JQ1 were specifically identified for our NPC subtypes, which may provide novel strategies for NPC therapy. Consistent with prior knowledge, we found that C1 patients were predicted to be sensitive to common therapeutic agents targeting cancer-related pathways such as PI3K, RTK and MAPK signaling (Table S6). For example, mitoxantrone, the most appropriate drug for C1 patients, was well-tolerated and produced an overall response rate for NPC in multicenter phase II trials [46]. We found that the medicines suitable for C2 patients were related to mitosis, genome integrity, and cell cycle pathways (Table S6). Evidence has shown the tight connections between the cell cycle, as well as mitosis and the pluripotency of stem cells [47]. Considering the stemness features of C2 patients, we provided several valuable drugs for NPC patients at high risk of disease recurrence and metastasis. Docetaxel has been reported to be effective in the metastatic or recurrent NPC patients who have failed at the first line of chemotherapy [48]. Moreover, as a semisynthetic derivative of vinblastine, vinorelbine has a moderately high activity and favorable toxicity profile for cisplatin-resistant patients in a phase II study [49]. For stem cell-based therapeutic regimens, Yu et al. found that PinX1 could inhibit the stemness of NPC by regulating the P53/miR-200b signal, thus suppressing the expression of Snail1, Twist1 and Zeb1 [40]. Notably, a lower IC50 value for telomerase inhibitor was observed in the C2 cluster, which was consistent with the function of PinX1 in NPC stemness. We further revealed the potential effects of telomerase inhibitor in NPC cells by the CCK-8 assay. Therefore, we indeed identified the chemotherapy drugs with potential applications in NPC treatment.

To prioritize essential genes associated with NPC stemness, we performed WGCNA and Cox regression analysis and filtered out PSMC3IP, NABP2, CDC45, and HJURP as potential targets. All of these genes were obtained from the blue module that positively correlated with the C2 cluster and stemness features. Moreover, the expression of these genes was upregulated in NPC tissues from multiple datasets and related to the poor survival of tumor patients, which indicated the potential of these genes as biomarkers in routine biopsy samples. The well-known stemness marker OCT4 has been found to facilitate stemness and radio resistance by regulating PSMC3IP, and the inhibition of PSMC3IP further reduced the self-renewal capacity of HNSC [50]. In addition, CDC45 has been reported as a target of small molecule compounds for HNSC via bioinformatic analysis [51,52]. We further verified the relationships between these genes and NPC stemness through in vitro experiments, and knockdown of PSMC3IP, NABP2, CDC45, and HJURP significantly inhibited the sphere formation ability of tumor cells; these results provided novel targets for NPC treatment.

Through the integration of bioinformatics and in vitro experiments, our study revealed tumor subtypes and provided stemness-relevant genes for NPC treatment. Although sufficient NPC datasets were enrolled for verification to support our findings, a mouse model and NPC samples from our center are lacking at present, which may slightly limit the applicability of the results. We are continuing to collect NPC samples and perform functional experiments to investigate the biological mechanisms of these genes on NPC stemness, TIME and treatment targets. In addition, the classification of NPC stemness-related subtypes was based on the transcriptomic signatures associated with dedifferentiation states in bulk gene expression data, rather than on the biological functionality of cancer stem cells. Further utilization of transcriptomic signatures obtained from cancer stem cells in NPC single-cell datasets may improve the accuracy of cancer subtype classification. Moreover, the relationship between NPC stemness subtypes and immunotherapy response needs to be validated in the future, since there is no public cohort of NPC patients receiving immunotherapy.

## 5. Conclusions

In conclusion, our study systematically characterized the stem cell landscape of NPC patients. The NPC subtypes we identified exhibited an inverse correlation between stemness status and immune features, which illustrates that stemness leads to an immune exclusion phenotype in the NPC TIME. We also screened several valuable drugs for NPC recurrence and metastasis. Through bioinformatics analysis and experimental verification, we determined that PSMC3IP, NABP2, CDC45, and HJURP are involved in NPC stemness, which will foster the development of therapeutic stem cell strategies for NPC patients.

## Data Availability

All data used in our study are publicly available. RNA-seq data and corresponding clinical information of NPC samples were obtained from GEO (https://www.ncbi.nlm.nih.gov/geo/, accessed on 24 October 2022) with accession ID GSE102349. Four microarrays of NPC and normal tissues were selected as external datasets (GSE12452, GSE13597, GSE53819, and GSE61218). The microarrays of NPC CSCs and non-CSCs determined by spheroid culture were collected from the GEO database with accession ID GSE36124. The transcriptome of head and neck squamous cell carcinoma (HNSC) was obtained from The Cancer Genome Atlas (TCGA, https://portal.gdc.cancer.gov/, accessed on 3 December 2022) portal. The software and tools applied in this article are described in Section 2. No special code was used in this study. Full pictures of the Western blots are presented in Figure S10.

## Supplementary Materials

The following supporting information can be downloaded at https://www.mdpi.com/article/10.3390/cancers18030422/s1. Figure S1. Box plots showing the activities of MsigDB stemness catogories estimated by ssGSEA in NPC subtypes. Figure S2. Clinical information of NPC subtypes. Figure S3. Scale independence and mean connectivity of multiple soft-thresholding powers (β) from 1 to 20 for WGCNA analysis. Figure S4. Functional enrichment results for the hub genes from. Figure S5. The expression patterns of PSMC3IP, NABP2, CDC45, and HJURP in NPC subtypes for GSE102349. Figure S6. The expression levels of PSMC3IP, CDC45, and HJURP in NPC non-CSC and CSC cells. Figure S7. The expression levels of PSMC3IP, HJURP, CDC45 in HNE1 and NABP2 in CNE2 cells transduced with siRNA. Figure S8. Effects of telomerase inhibitor on NPC cell proliferation. Figure S9. The TIME, immunotherapy response, and drug sensitive for original NPC clusters. Figure S10. Original images of Western Blot. Table S1. The characteristic of datasets in this study. Table S2. Stemness gene sets used in this study. Table S3. Primer of genes for RT-qPCR. Table S4. siRNA sequences used in this study. Table S5. Differently expressed genes in C1 cluster compared to C2 cluster. Table S6. Literatures for the drugs reported for NPC therapy.

## Abbreviations

The following abbreviations are used in this manuscript:

NPC	nasopharyngeal carcinoma
CSCs	cancer stem cells
TIME	tumor immune microenvironment
WGCNA	weighted gene correlation network analysis
OCLR	one-class logistic regression machine learning algorithm
PFS	progression-free survival
MsigDB	Molecular Signature Database
ssGSEA	single-sample GSEA
HLA	human leukocyte antigen
TILS	tumor-infiltrating lymphocytes
IC50	half-maximum inhibitory concentration
GDSC	Genomics of Drug Sensitivity in Cancer
TIDE	Tumor Immune Dysfunction and Exclusion
