# Supplementary material for: Comprehensive Characterization of Stem Cell Landscape Identifies Novel Stemness-Relevant Genes for Nasopharyngeal Carcinoma Therapy
Source: Cancers (Basel). 2026 Jan 28;18(3):422. doi: 10.3390/cancers18030422 (PMC12896439; doi:10.3390/cancers18030422)

Figure 6B, rep1:

PSMC3IP

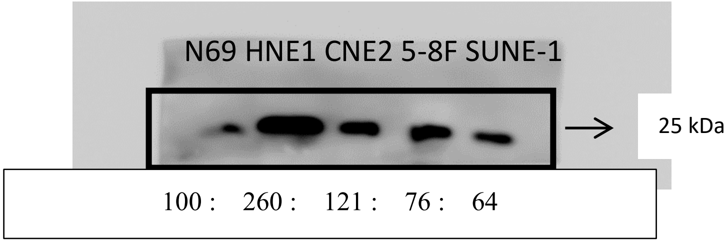

HJURP

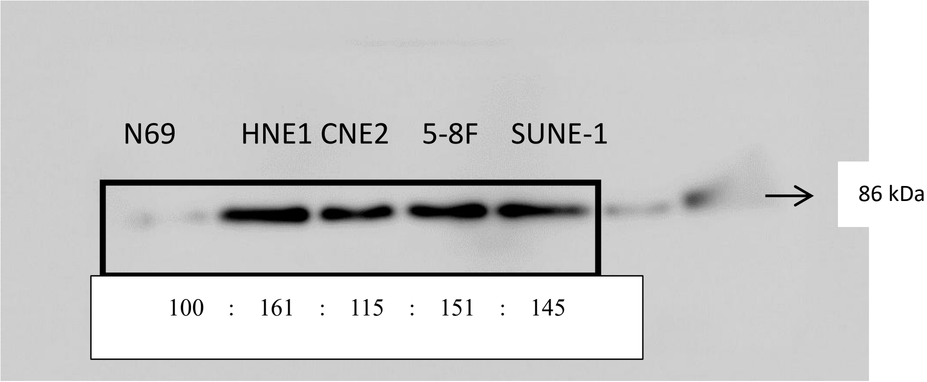

CDC45

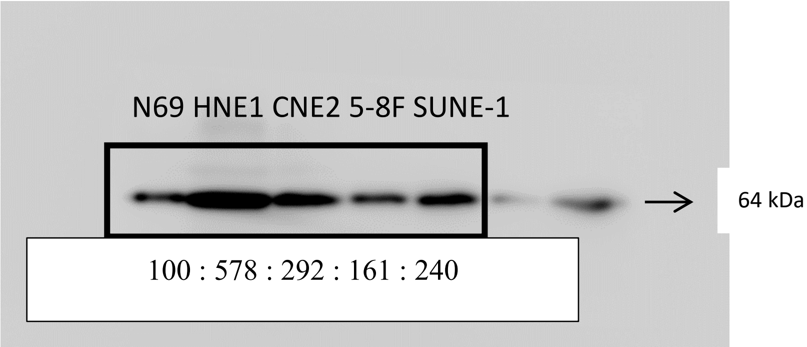

NABP2

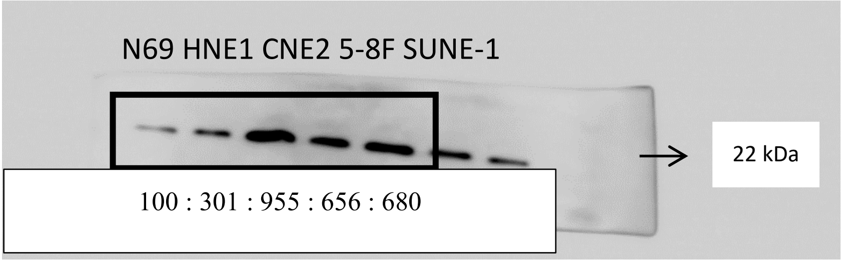

GAPDH

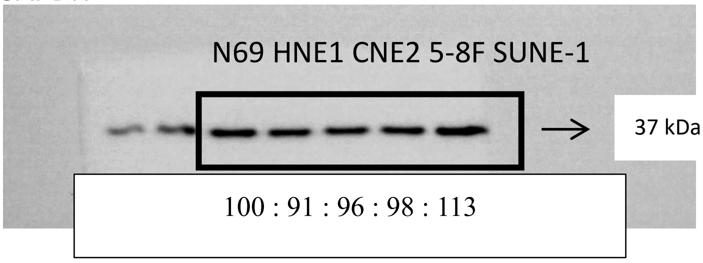

Figure 6B rep2:

PSMC3IP

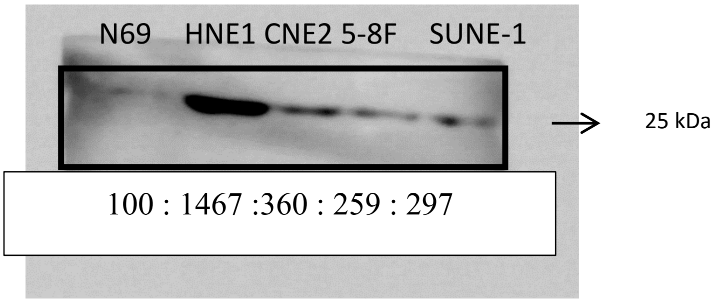

HJURP

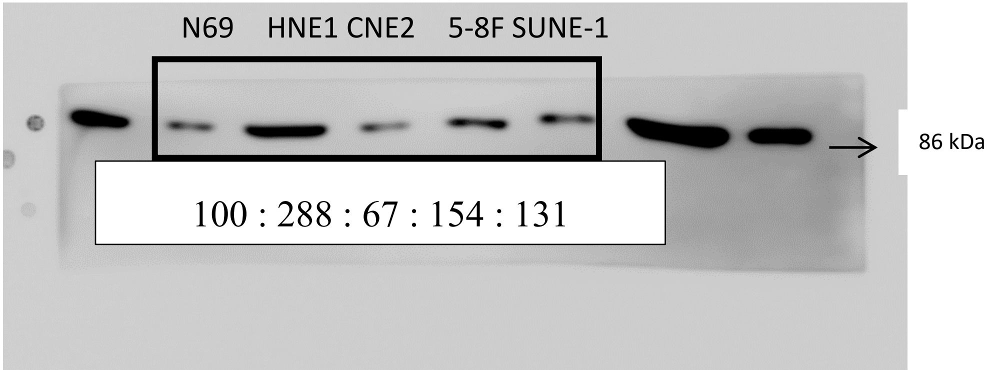

CDC45

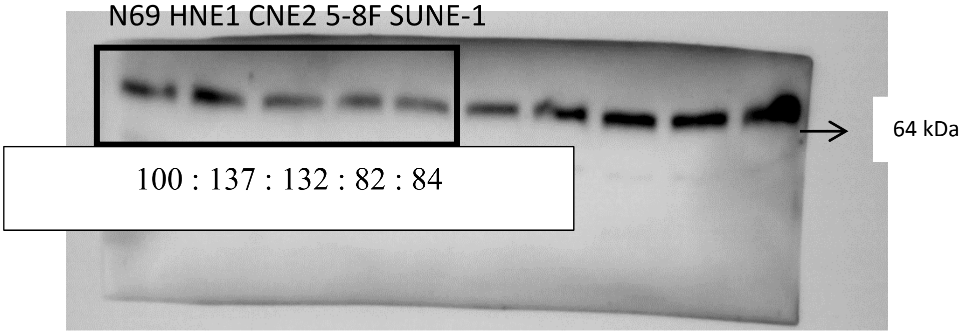

NABP2

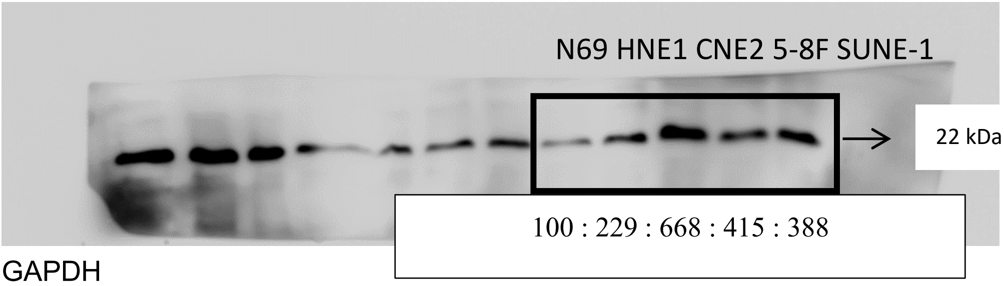

GAPDH

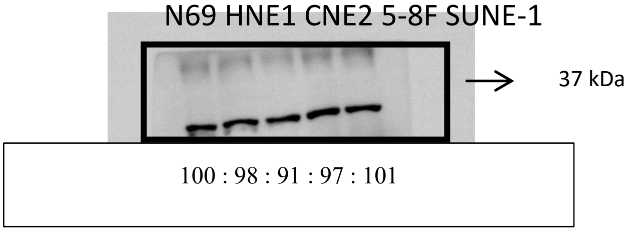

Figure 6B rep3:

PSMC3IP

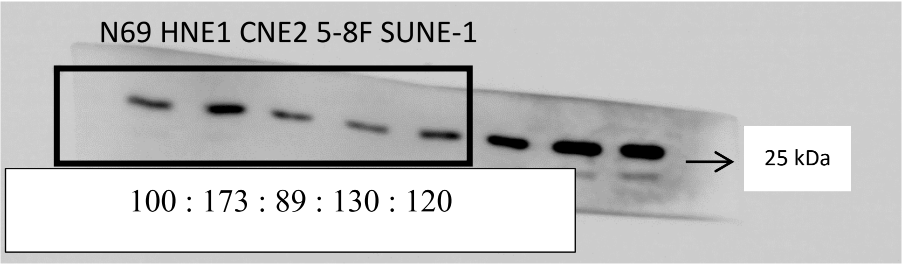

HJURP

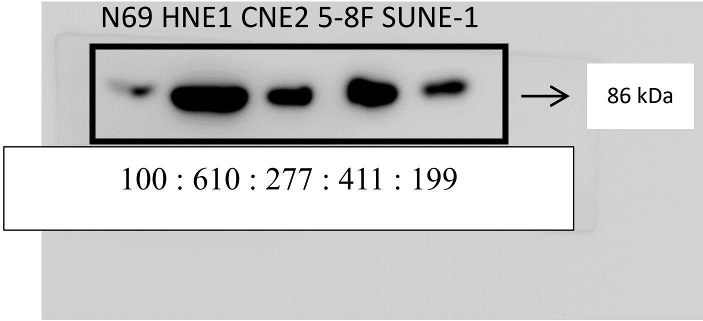

CDC45

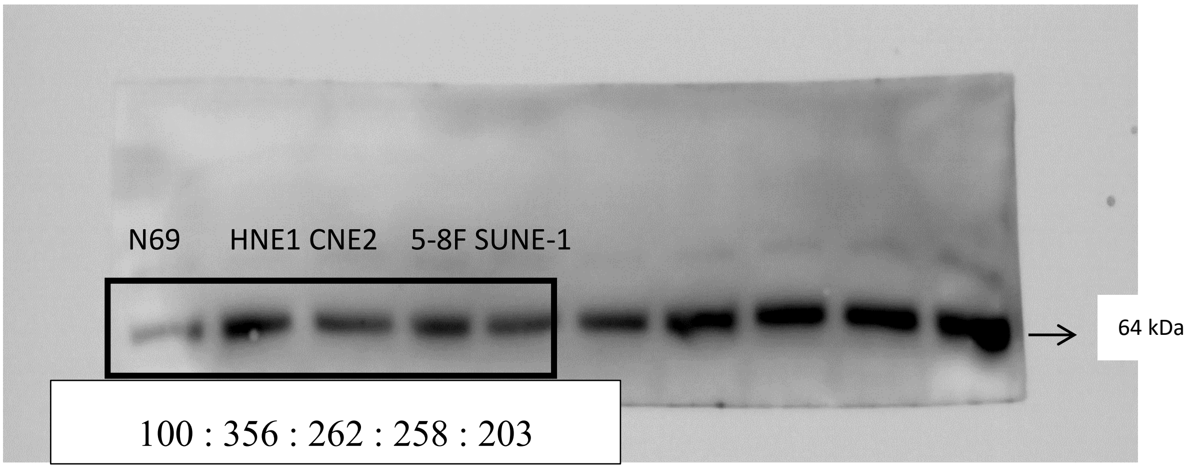

NABP2

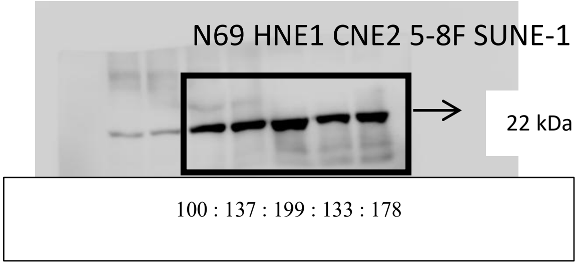

GAPDH

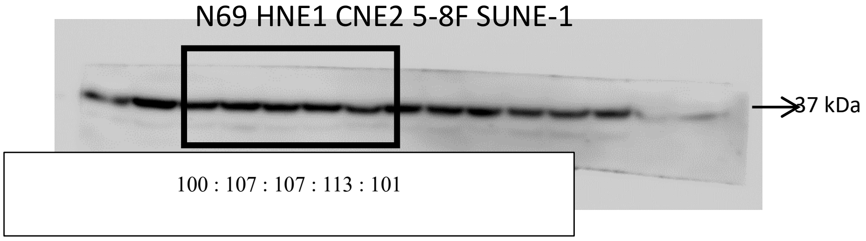

Figure 6C:

PSMC3IP rep1:

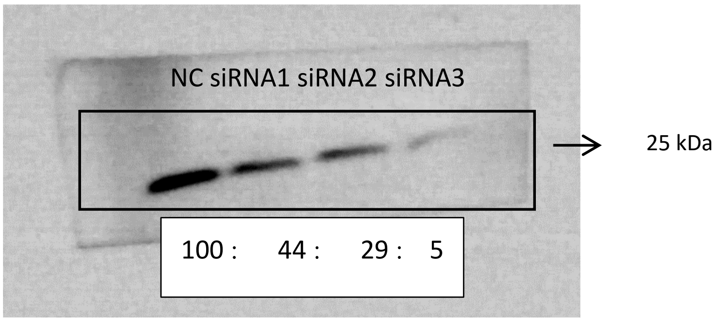

PSMC3IP rep2:

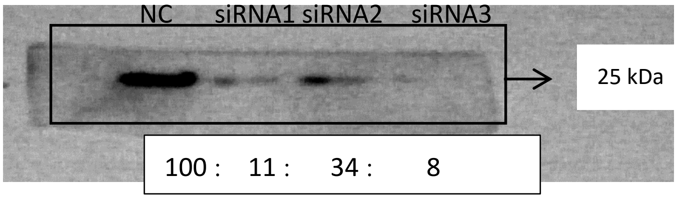

PSMC3IP rep3:

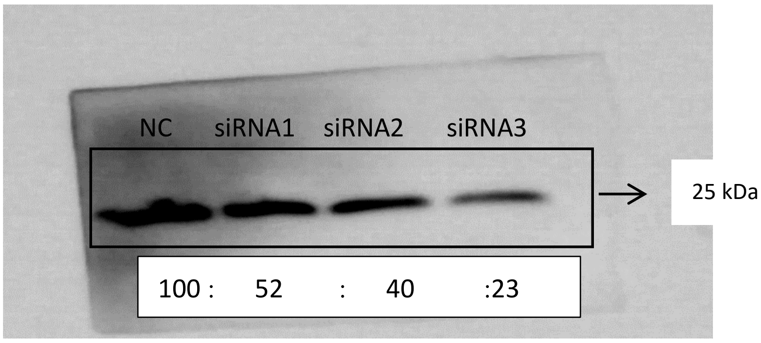

GAPDH rep1:

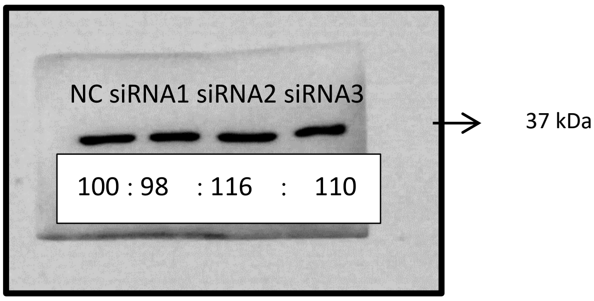

GAPDH rep2:

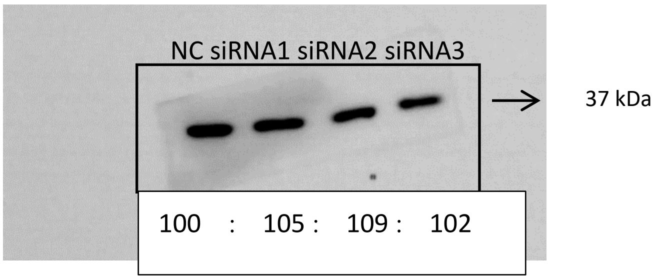

GAPDH rep3:

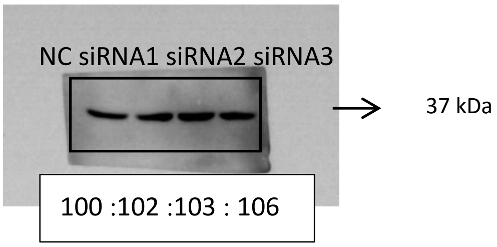

NABP2 rep1:

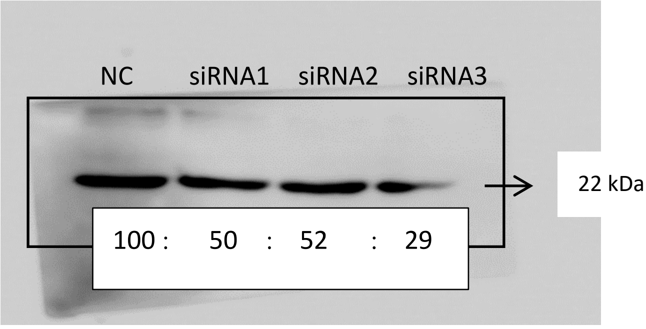

NABP2 rep2:

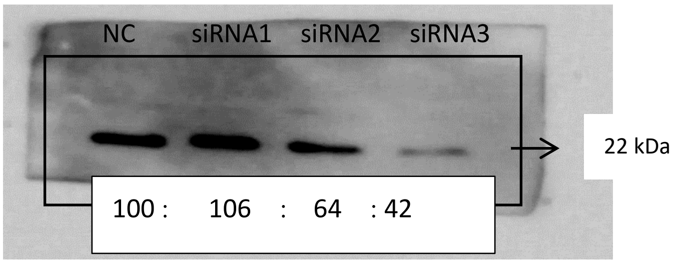

NABP2 rep3:

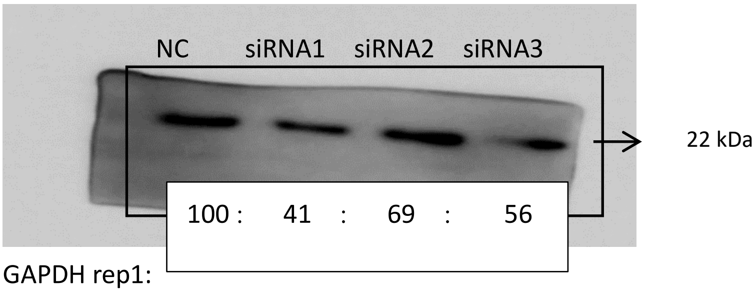

GAPDH rep1:

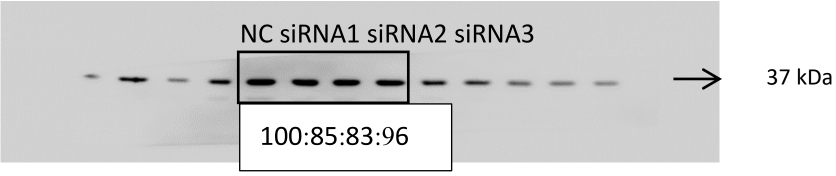

GAPDH rep2:

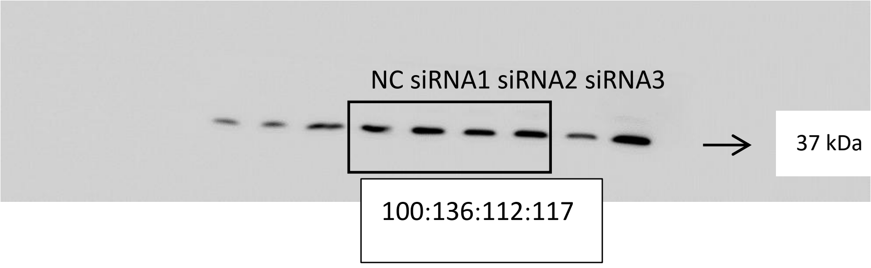

GAPDH rep3:

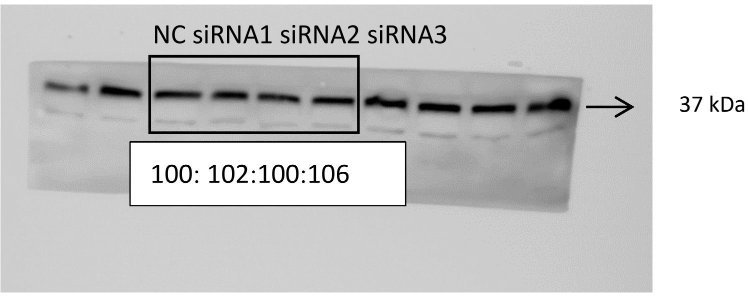

CDC45 rep1:

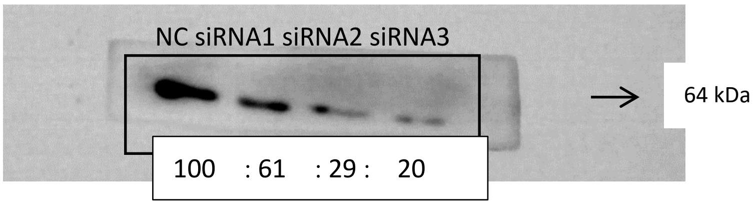

CDC45 rep2:

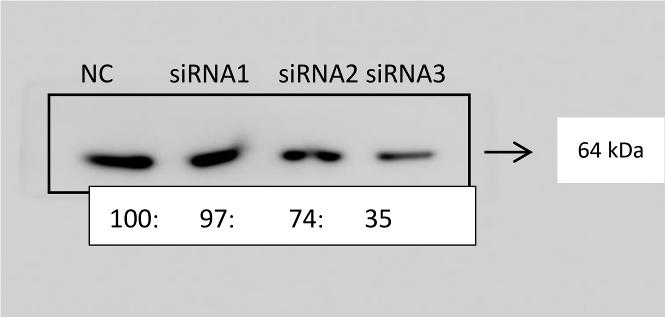

CDC45 rep3:

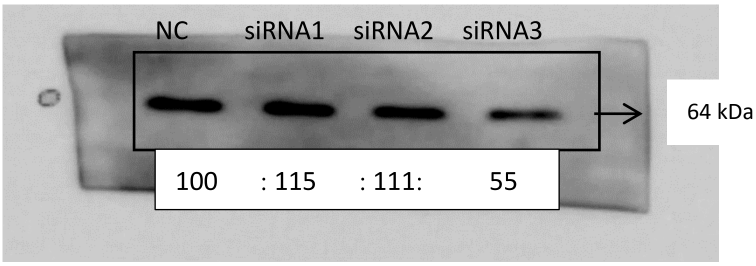

GAPDH rep1:

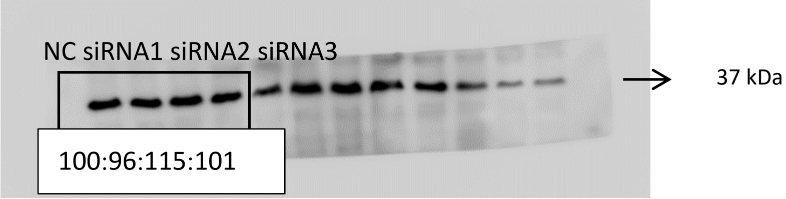

GAPDH rep2:

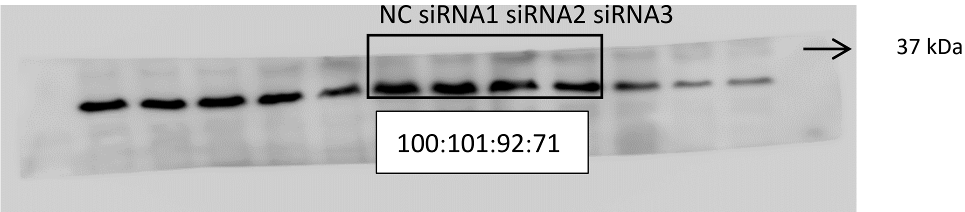

GAPDH rep3:

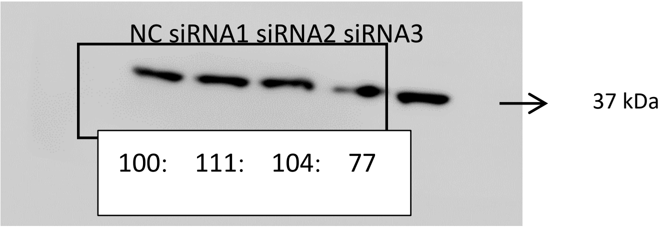

HJURP rep1:

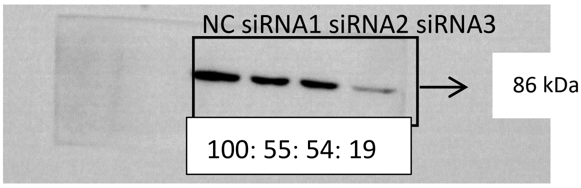

HJURP rep2:

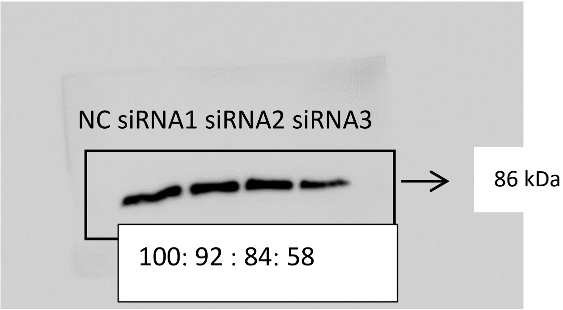

HJURP rep3:

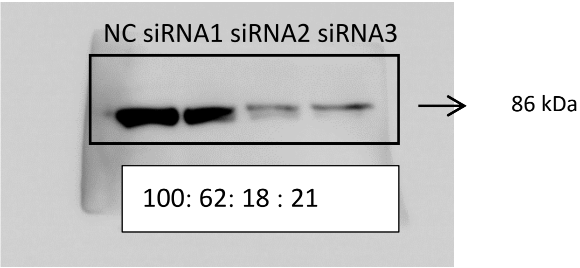

GAPDH rep1:

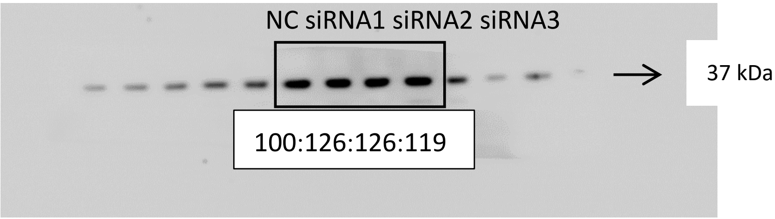

GAPDH rep2:

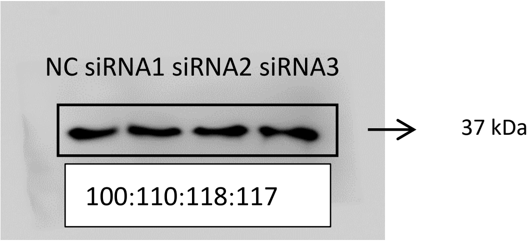

GAPDH rep3:

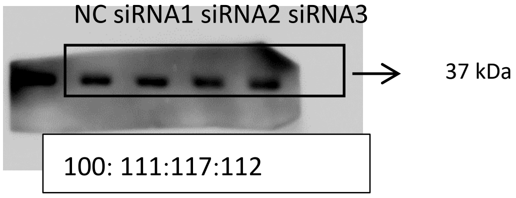

Figure 6E siCDC45:

LGR5 rep1:

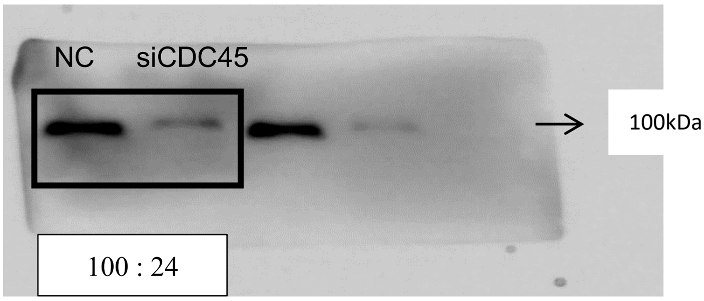

LGR5 rep2:

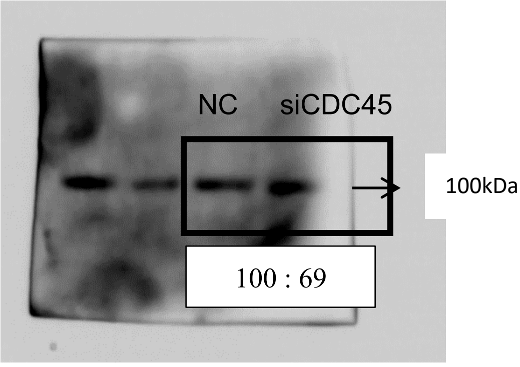

LGR5 rep3:

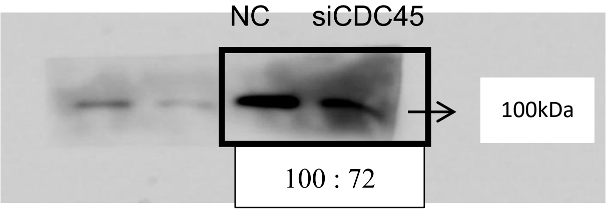

CD44 rep1:

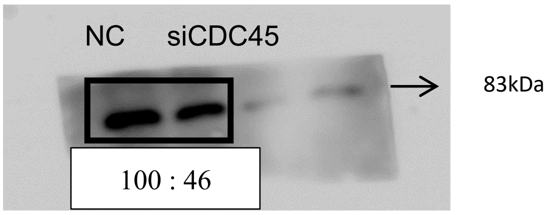

CD44 rep2:

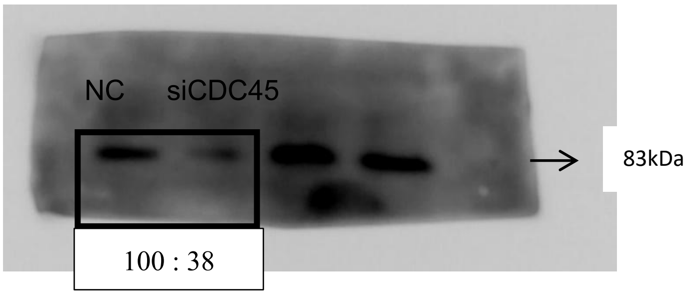

CD44 rep3:

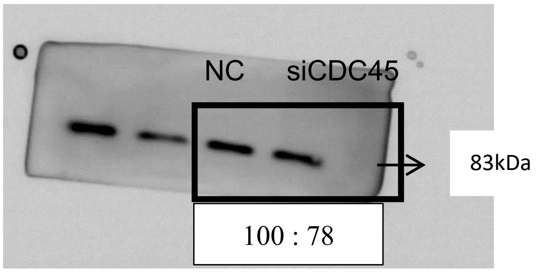

SOX2 rep1:

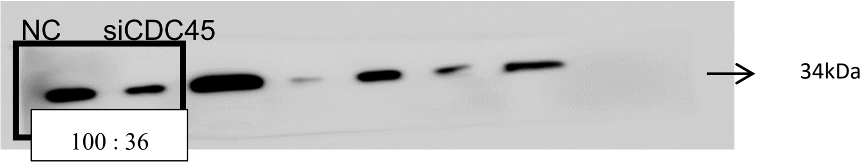

SOX2 rep2:

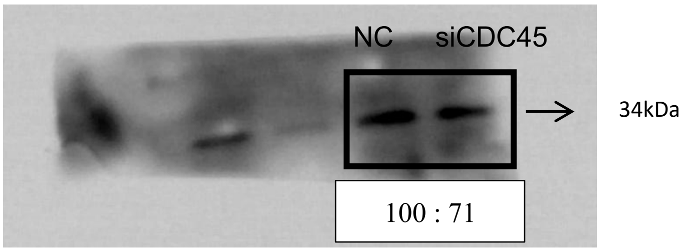

SOX2 rep3:

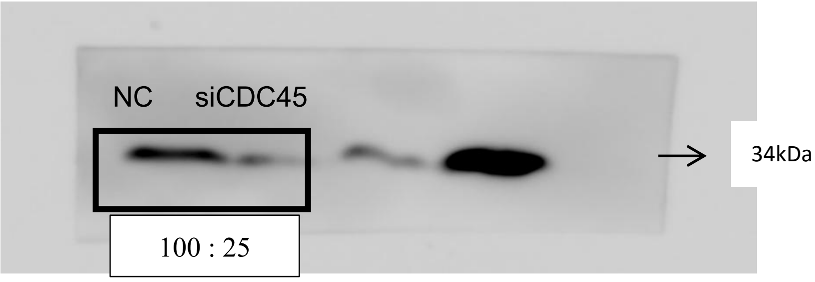

NANOG rep1:

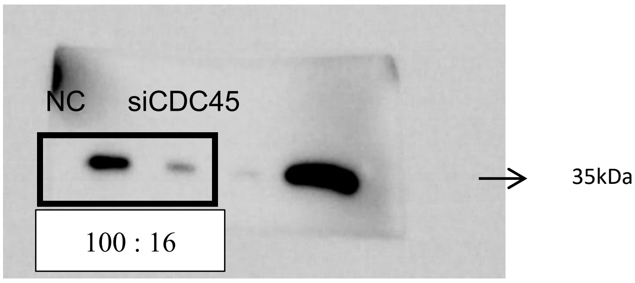

NANOG rep2:

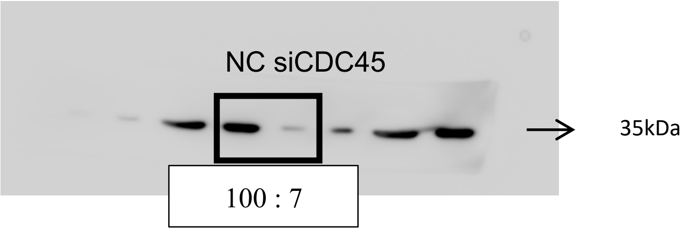

NANOG rep3:

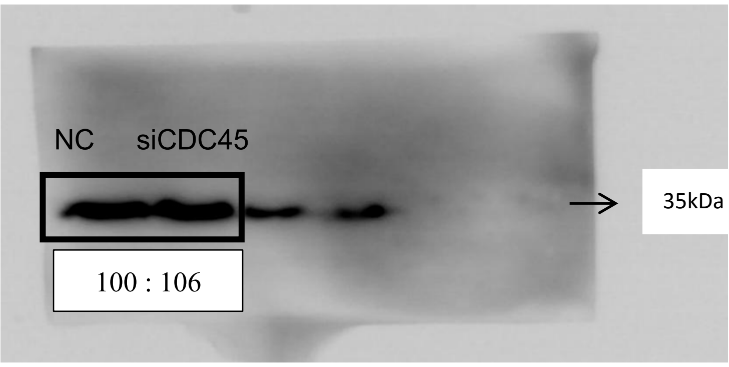

OCT4 rep1:

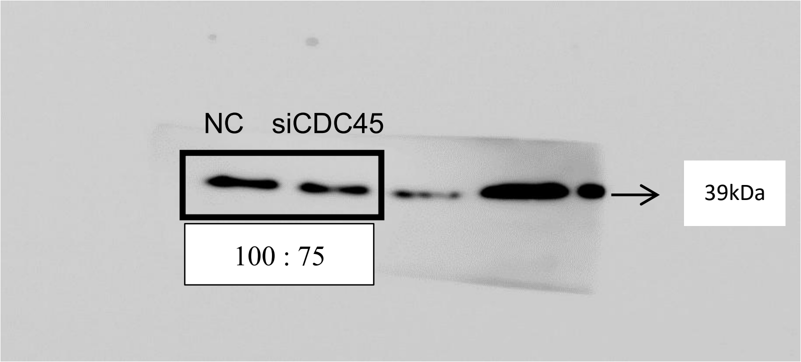

OCT4 rep2:

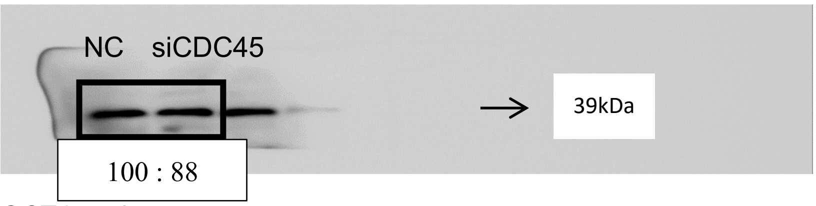

OCT4 rep3:

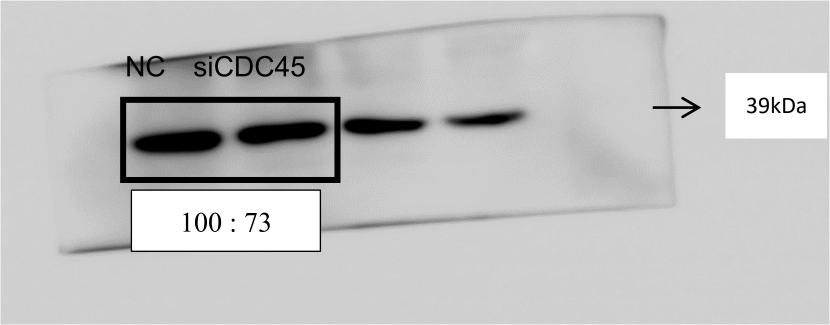

GAPDH rep1,2:

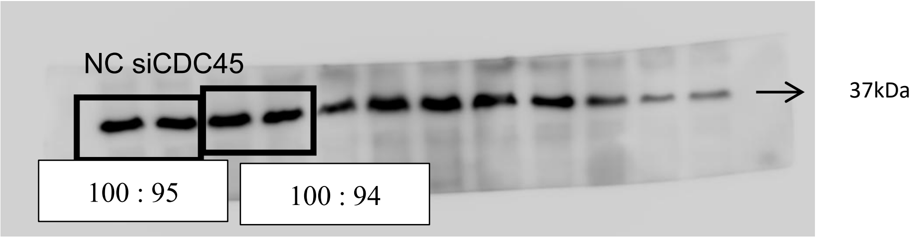

GAPDH rep3:

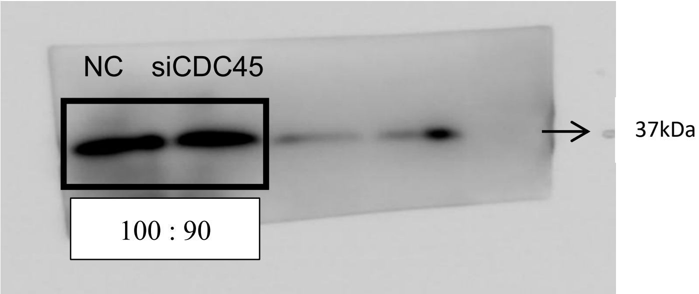

Figure 6E siHJURP:

LGR5 rep1:

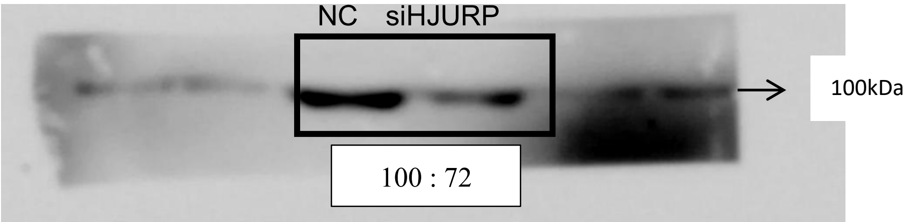

LGR5 rep2:

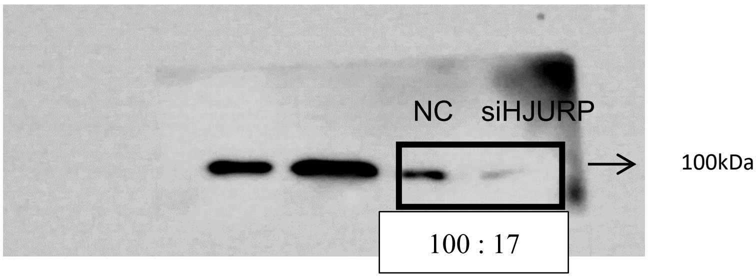

LGR5 rep3:

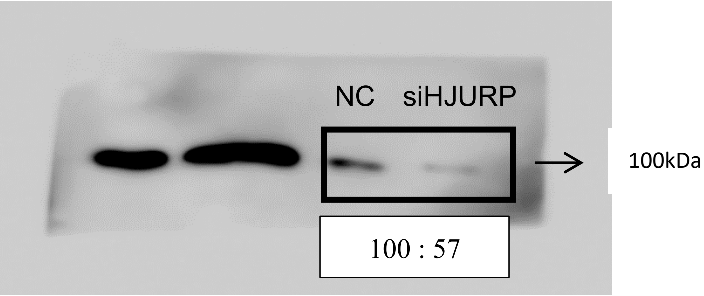

CD44 rep1:

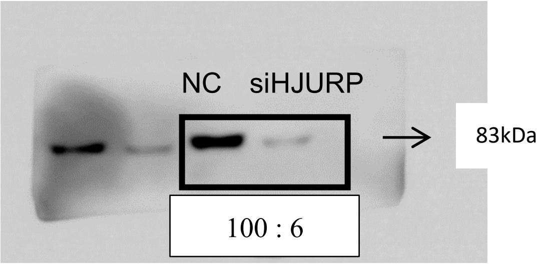

CD44 rep2:

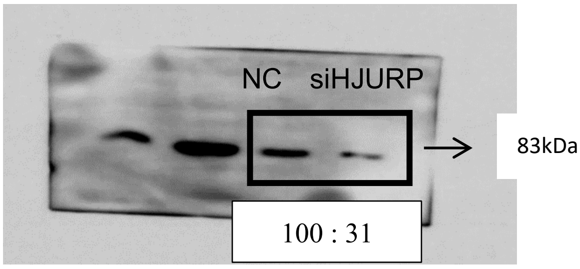

CD44 rep3:

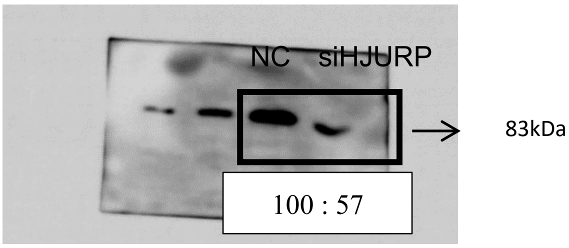

SOX2 rep1:

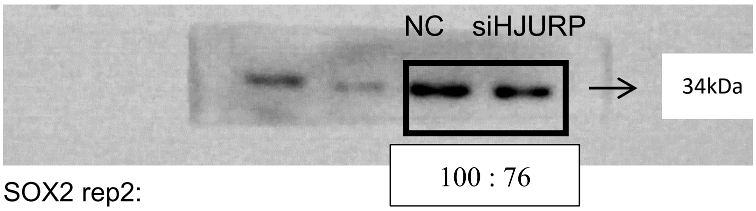

SOX2 rep2:

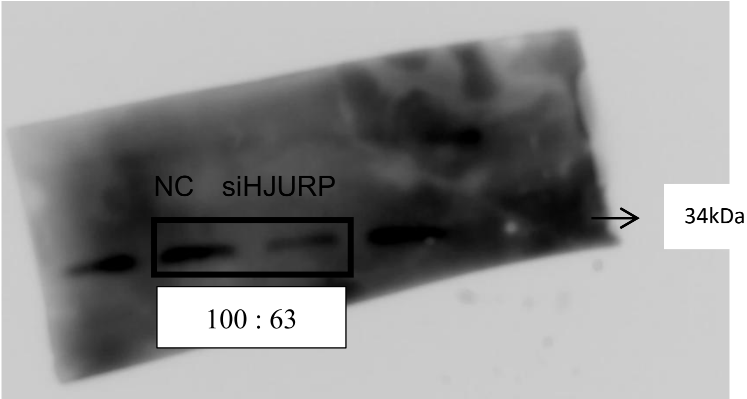

SOX2 rep3:

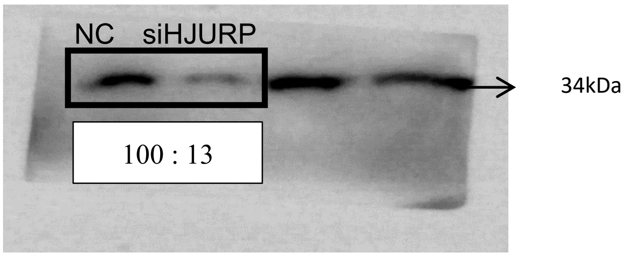

NANOG rep1:

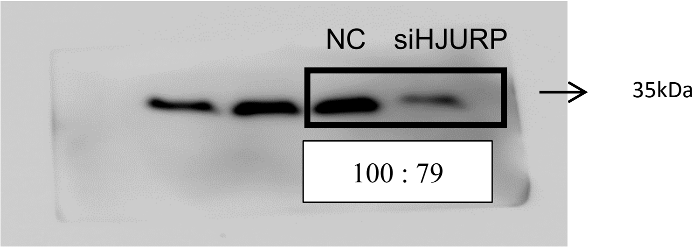

NANOG rep2:

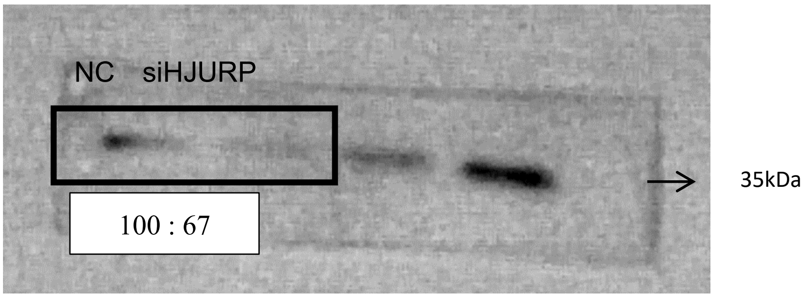

NANOG rep3:

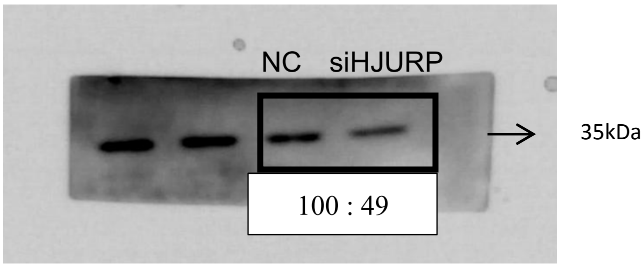

OCT4 rep1:

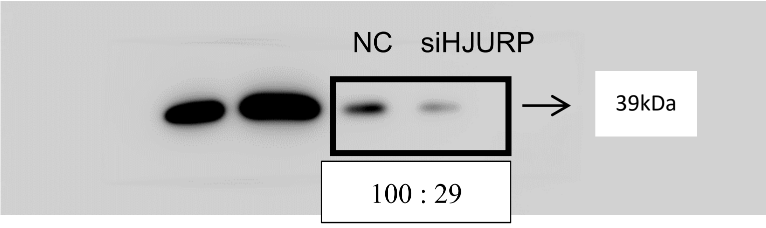

OCT4 rep2:

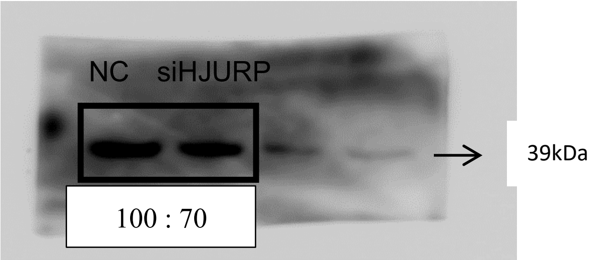

OCT4 rep3:

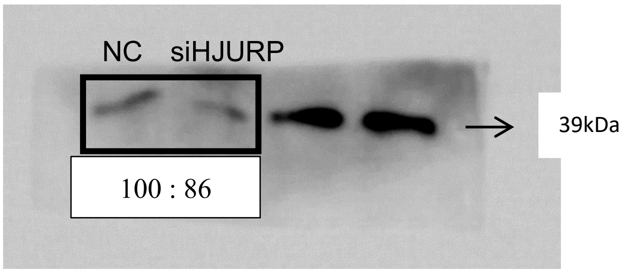

GAPDH rep1:

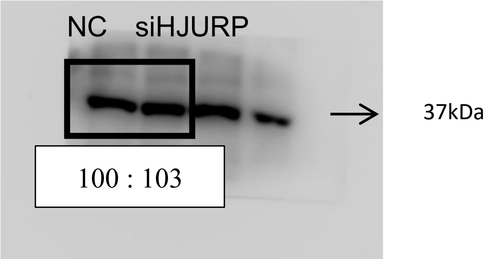

GAPDH rep2:

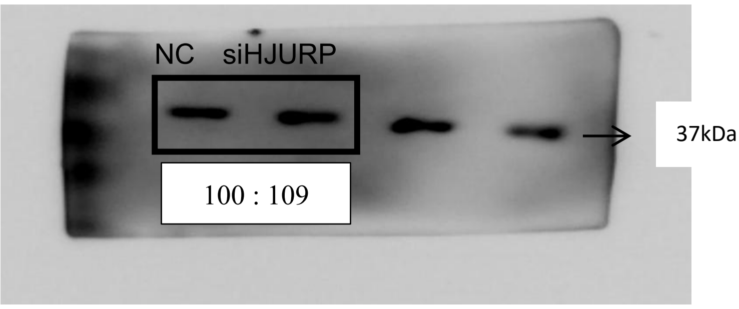

GAPDH rep3:

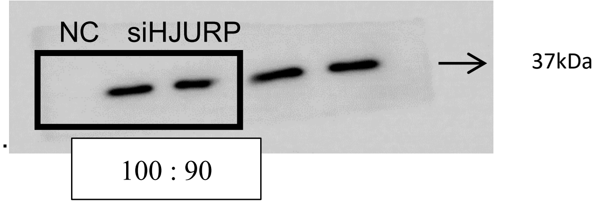

Figure 6E siNABP2:

LGR5 rep1:

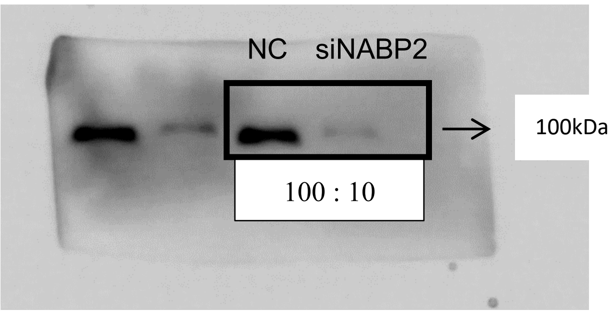

LGR5 rep2:

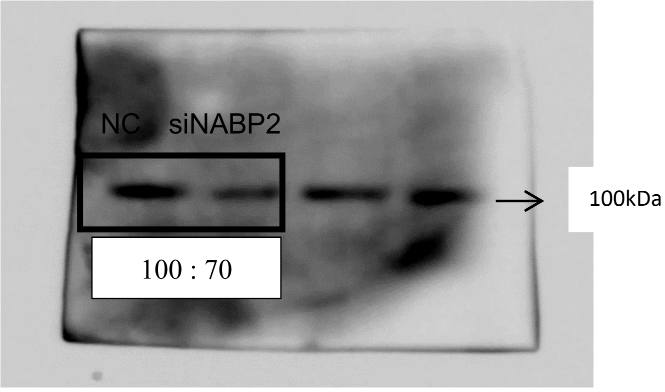

LGR5 rep3:

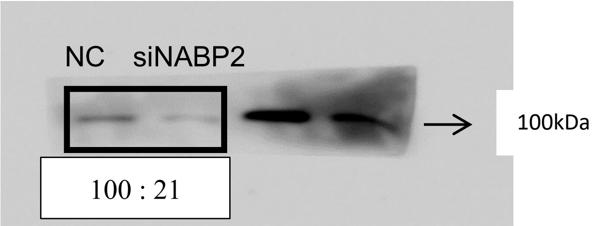

CD44 rep1:

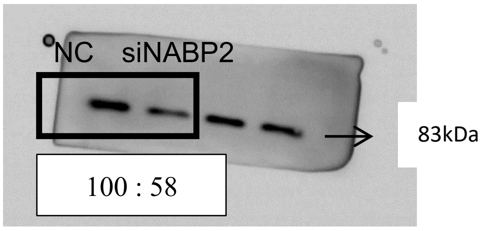

CD44 rep2:

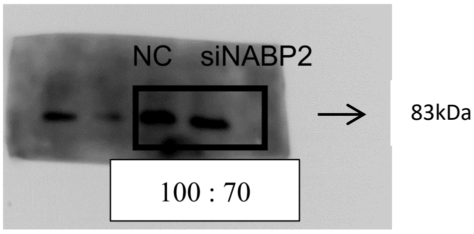

CD44 rep3:

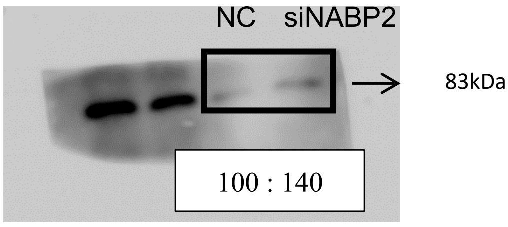

SOX2 rep1:

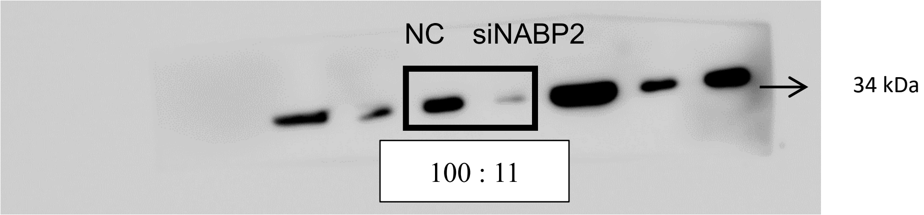

SOX2 rep2:

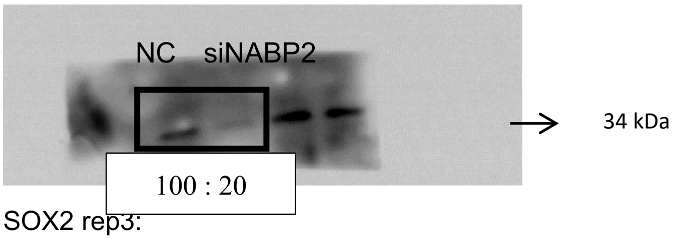

SOX2 rep3:

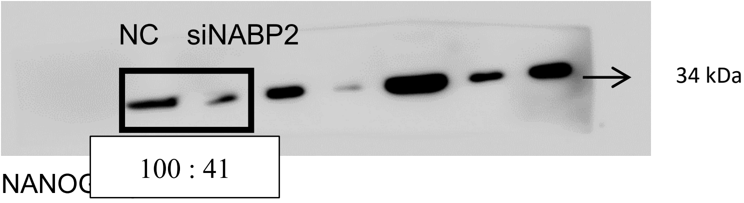

NANO[illegible]

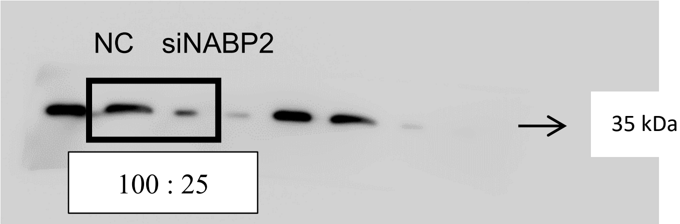

NANOG rep2:

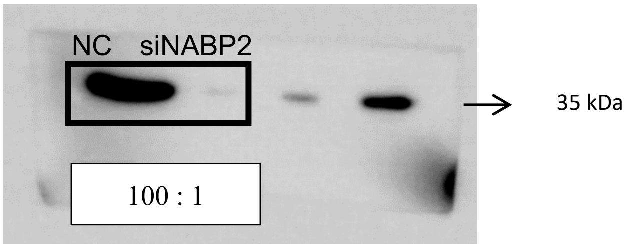

NANOG rep3:

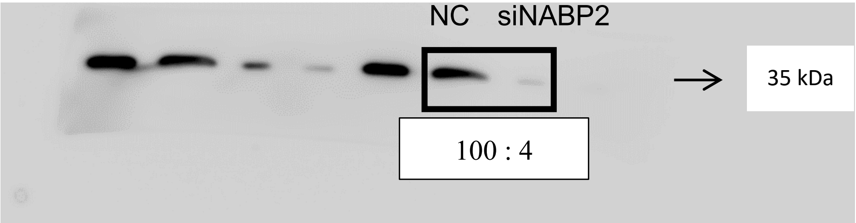

OCT4 rep1:

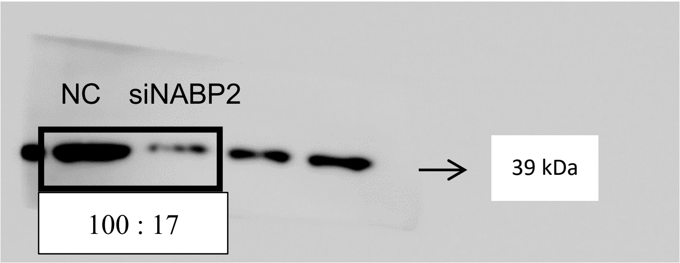

OCT4 rep2:

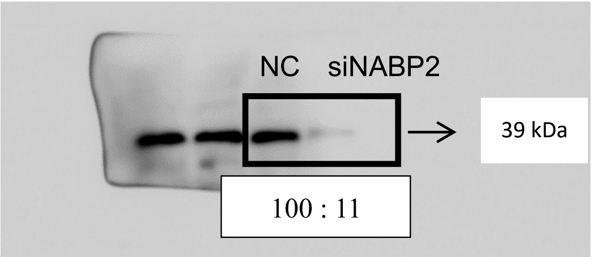

OCT4 rep3:

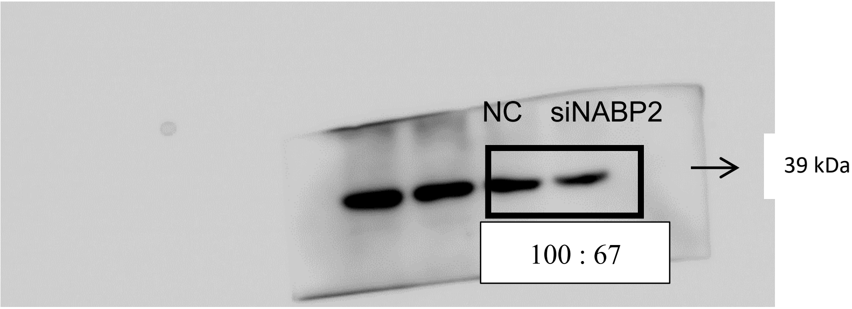

GAPDH rep1:

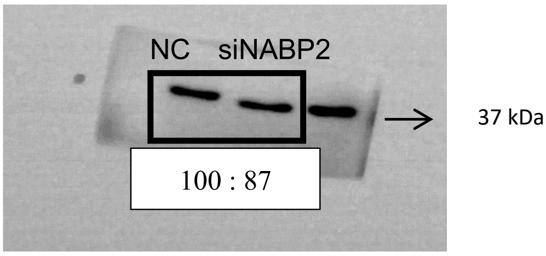

GAPDH rep2:

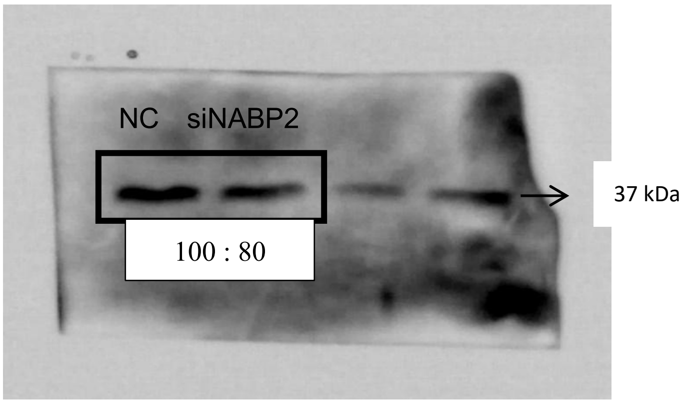

GAPDH rep3:

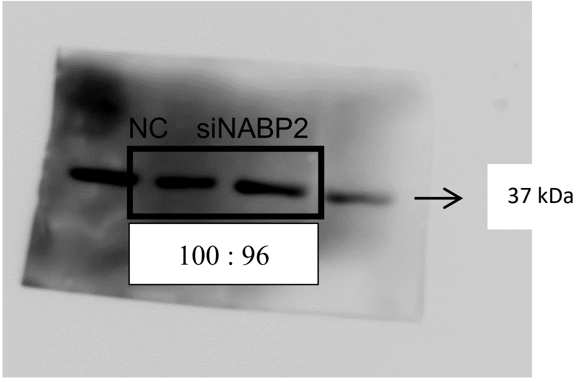

Figure 6E siPSMC3IP:

LGR5 rep1:

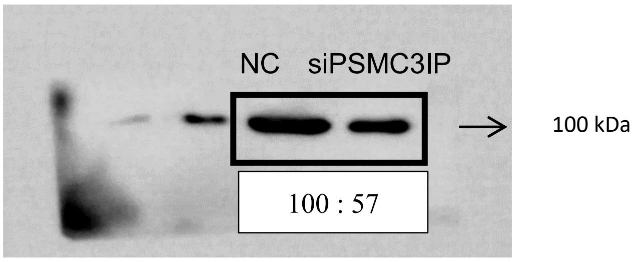

LGR5 rep2:

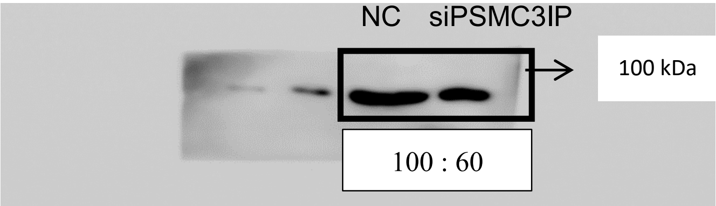

LGR5 rep3:

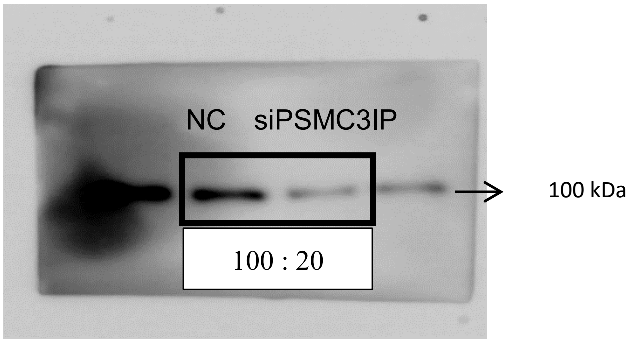

CD44 rep1:

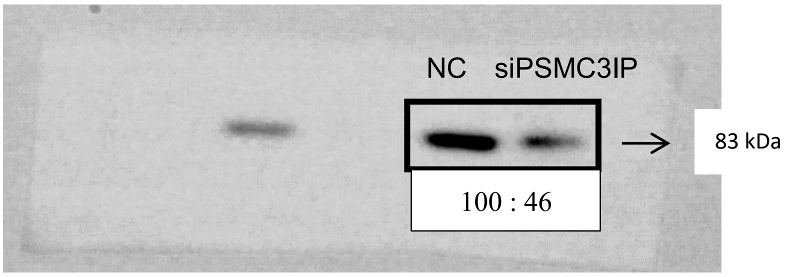

CD44 rep2:

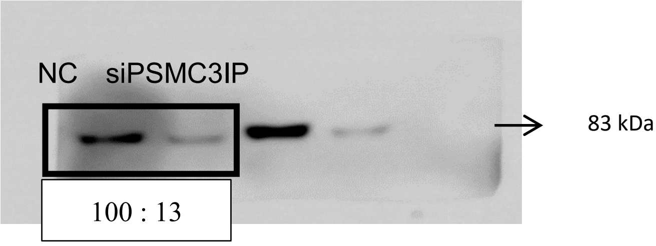

CD44 rep3:

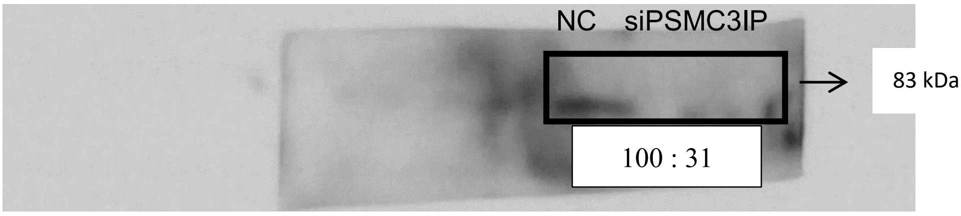

SOX2 rep1:

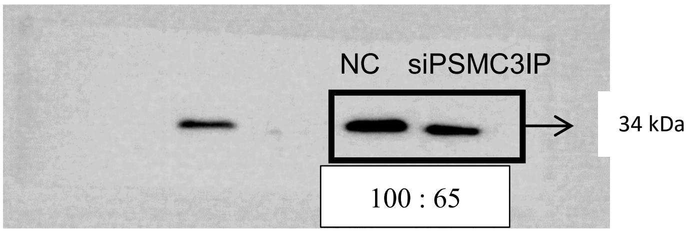

SOX2 rep2:

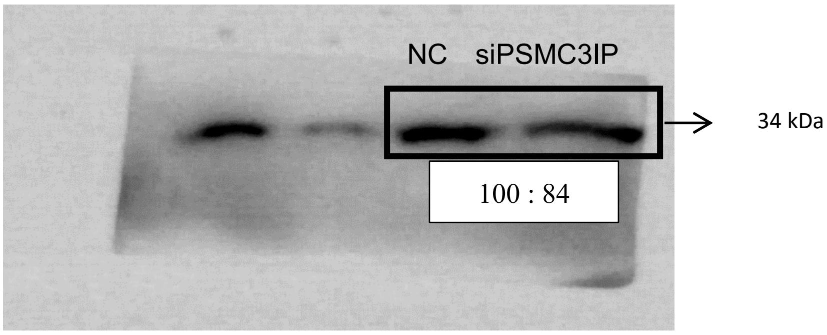

SOX3 rep3:

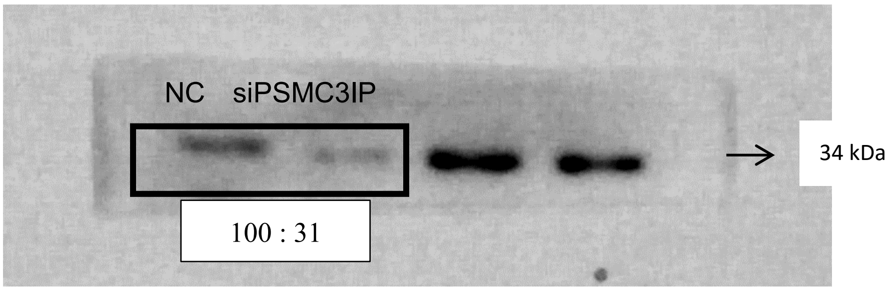

NANOG rep1:

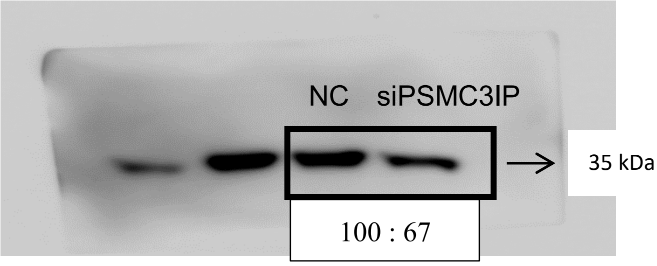

NANOG rep2:

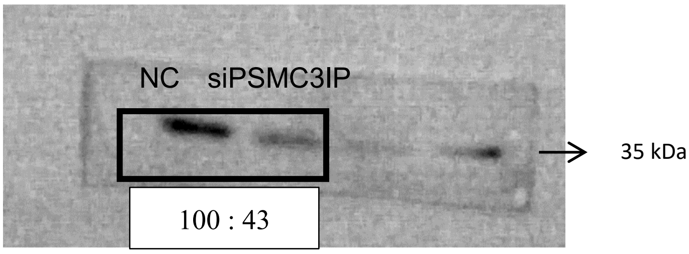

NANOG rep3:

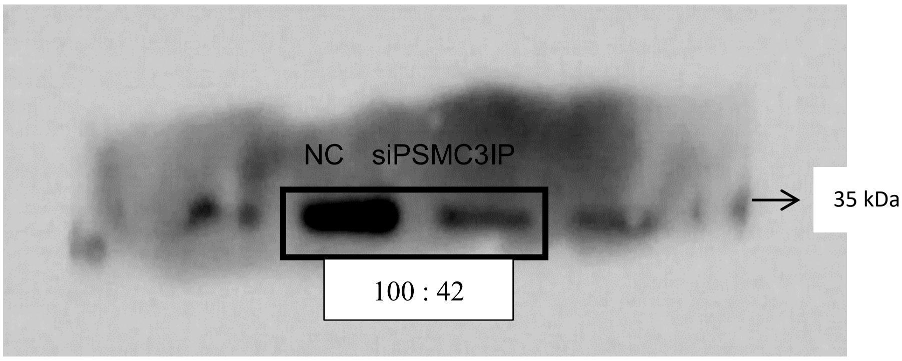

OCT4 rep1:

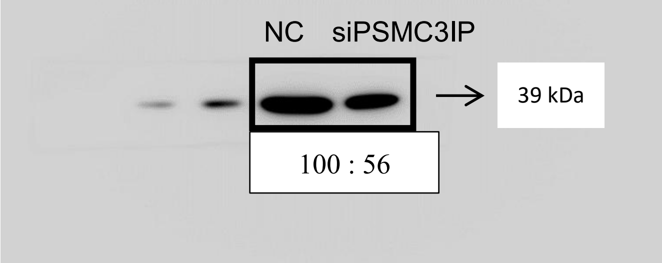

OCT4 rep2:

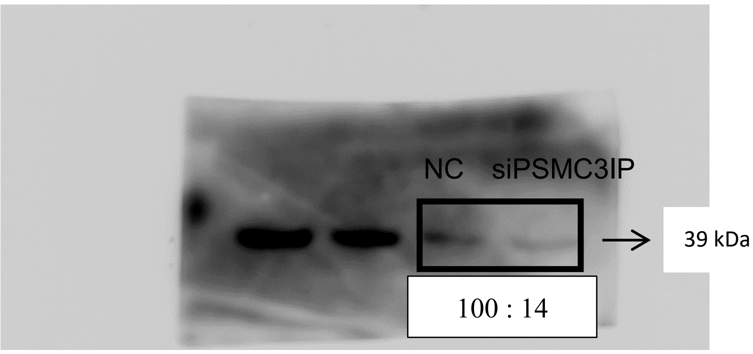

OCT4 rep3:

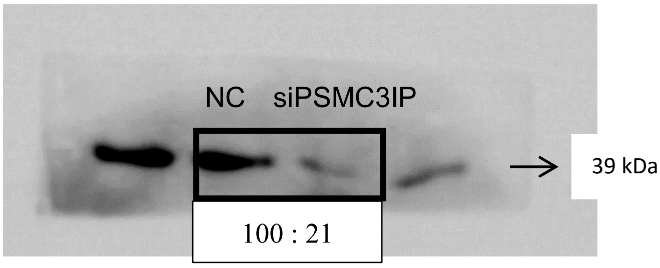

GAPDH rep1:

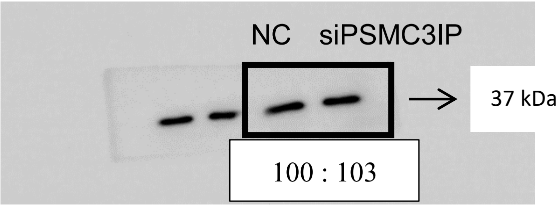

GAPDH rep2:

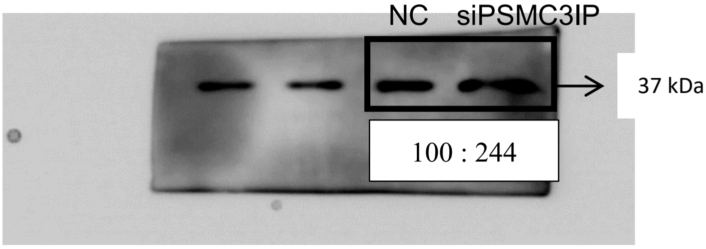

GAPDH rep3:

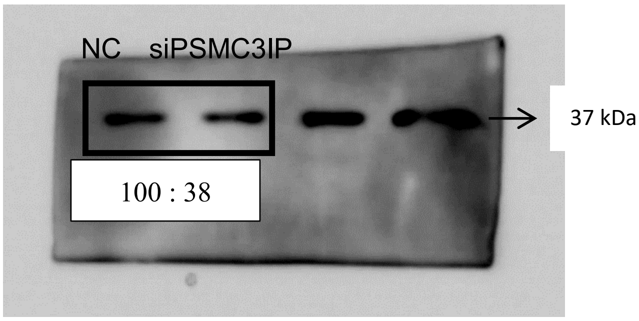

Supplement: Supplementary file 1 [file cancers-18-00422-s001.zip › Supplementary figure S10_Original images of Western Blot.pdf]
