# Supplementary material for: Comprehensive Characterization of Stem Cell Landscape Identifies Novel Stemness-Relevant Genes for Nasopharyngeal Carcinoma Therapy
Source: Cancers (Basel). 2026 Jan 28;18(3):422. doi: 10.3390/cancers18030422 (PMC12896439; doi:10.3390/cancers18030422)
Supplement: Supplementary file 1 [file cancers-18-00422-s001.zip › Supplementary figure S1-S9.pdf]

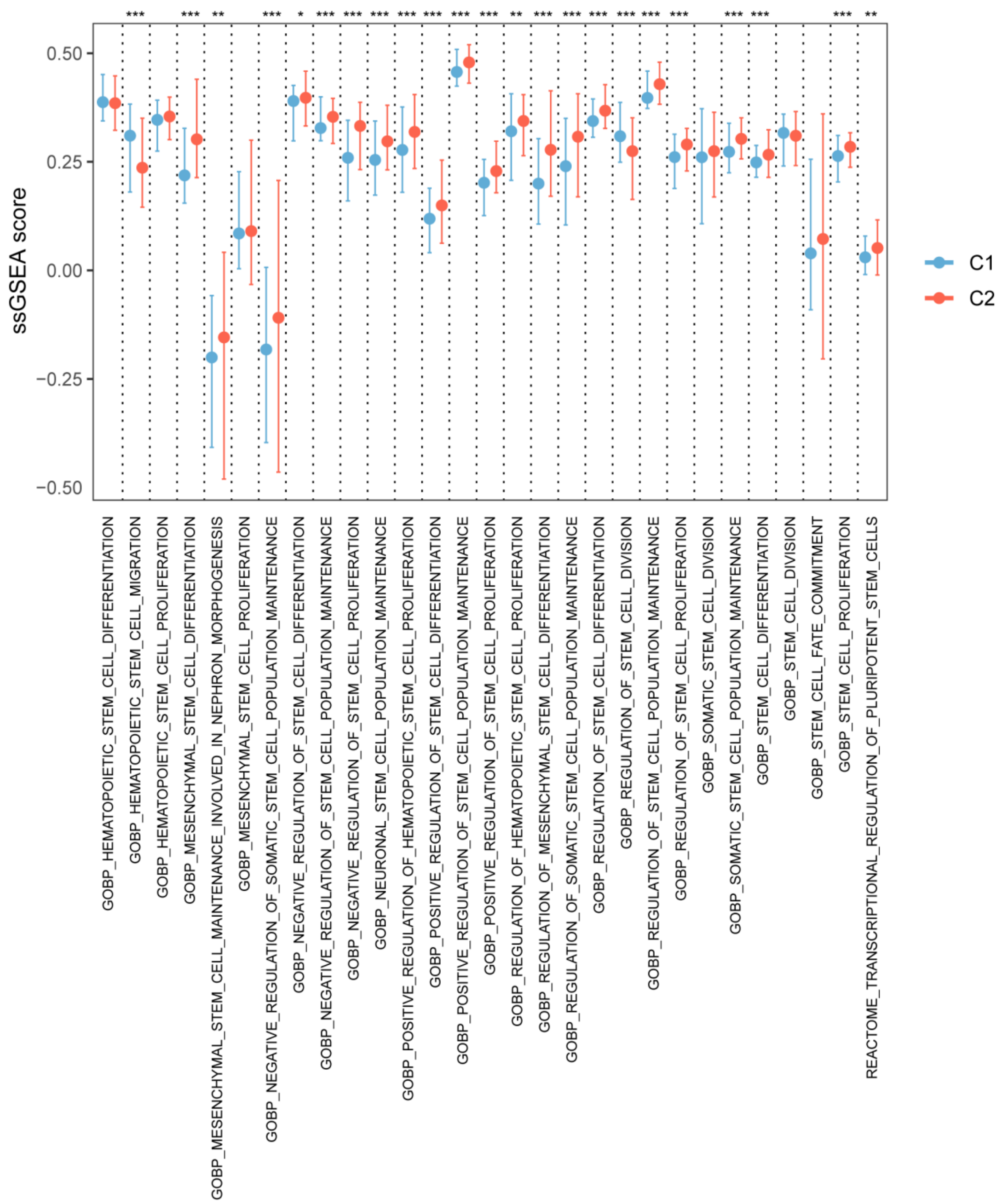

Figure S1. Box plots showing the activities of MsigDB stemness catogories estimated by ssGSEA in NPC subtypes.

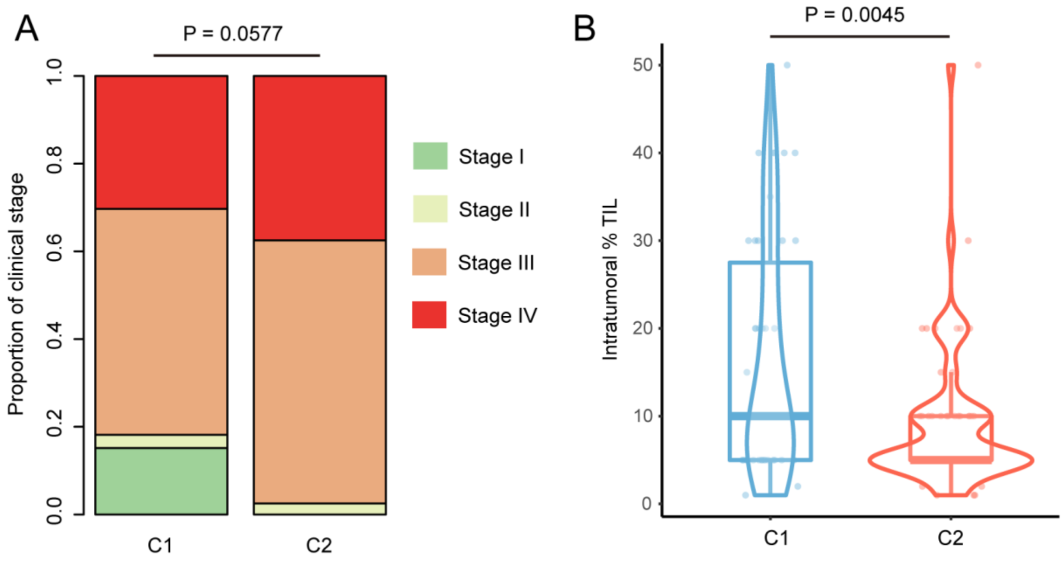

Figure S2. Clinical information of NPC subtypes. (A) Bar plots showing the proportion of cancer stages in each NPC cluster. (B) The difference of intratumoral TILs between NPC subtypes.

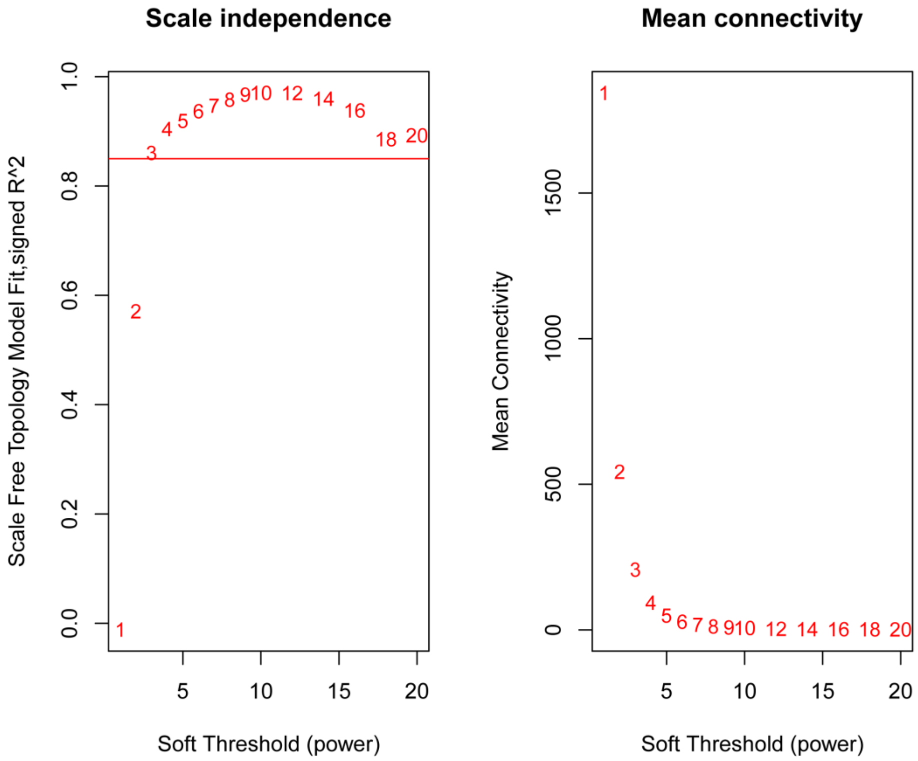

Figure S3. Scale independence and mean connectivity of multiple soft-thresholding powers (β) from 1 to 20 for WGCNA analysis.

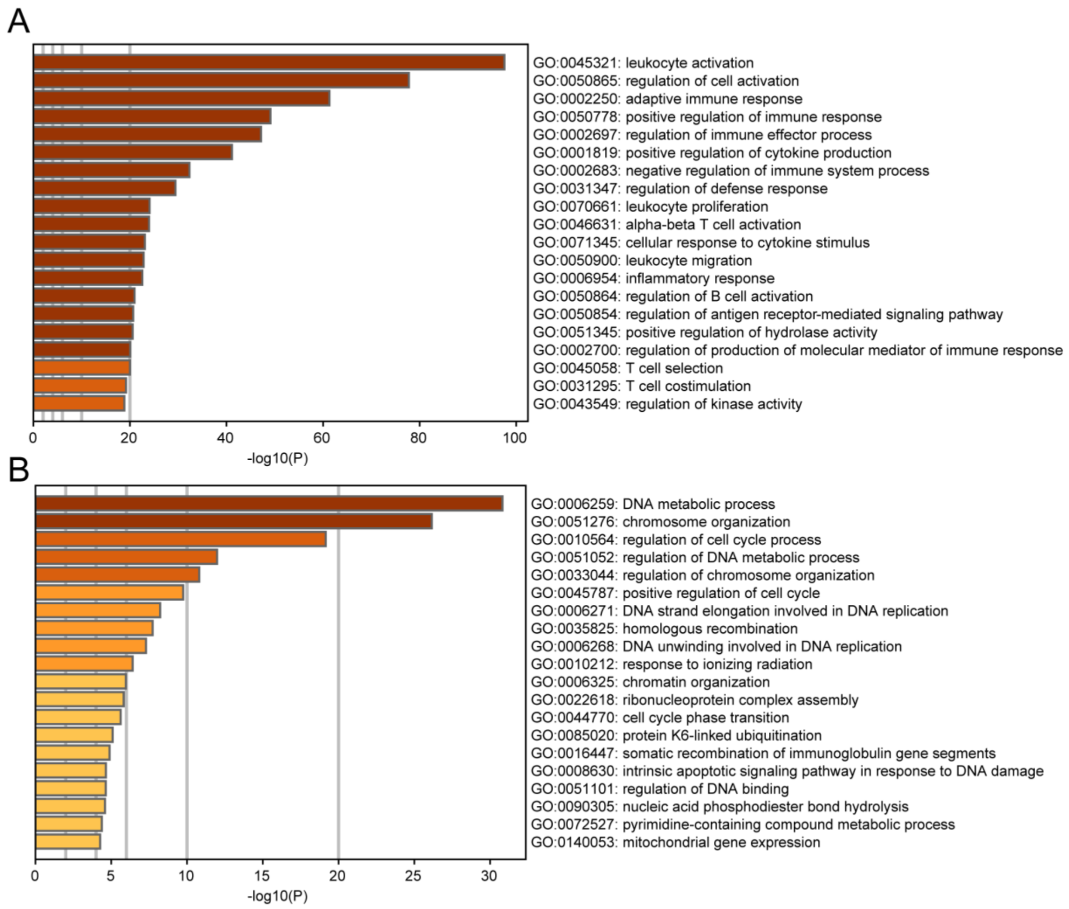

Figure S4. Functional enrichment results for the hub genes from (A) turquoise and (B) blue modules.

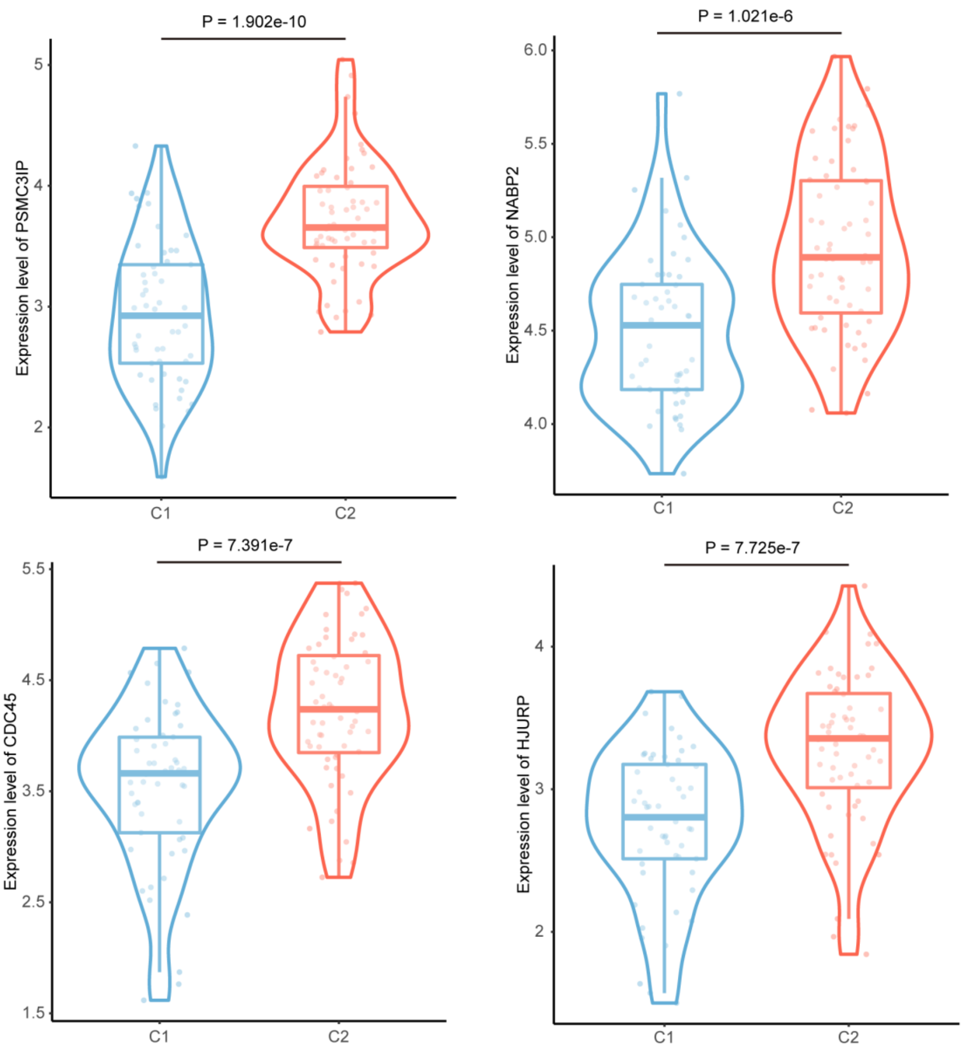

Figure S5. The expression patterns of PSMC3IP, NABP2, CDC45, and HJURP in NPC subtypes for GSE102349.

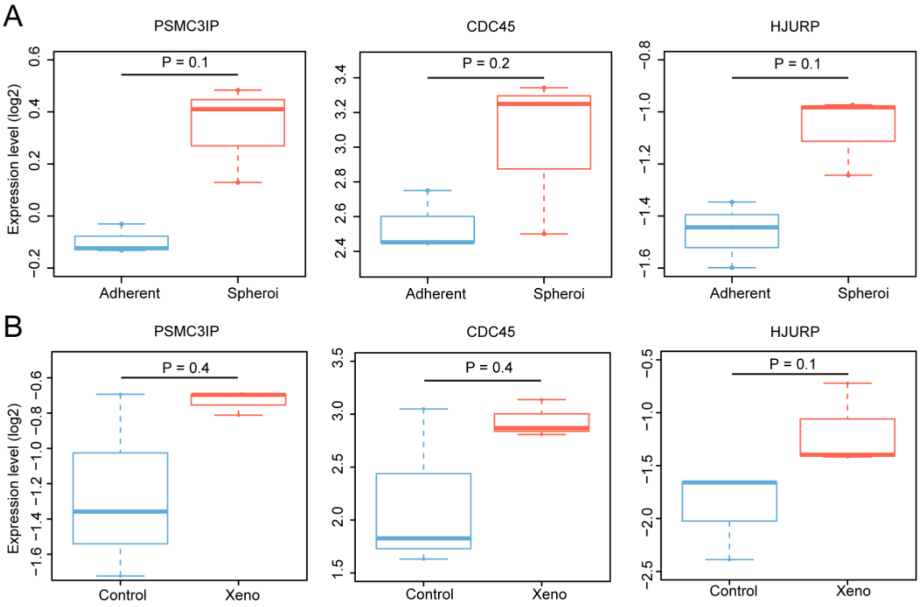

Figure S6. The expression levels of PSMC3IP, CDC45, and HJURP in NPC non-CSC and CSC cells. (A) spheroid culture and (B) stem-like cells inoculated into immunocompromised mice for xenograft tumors.

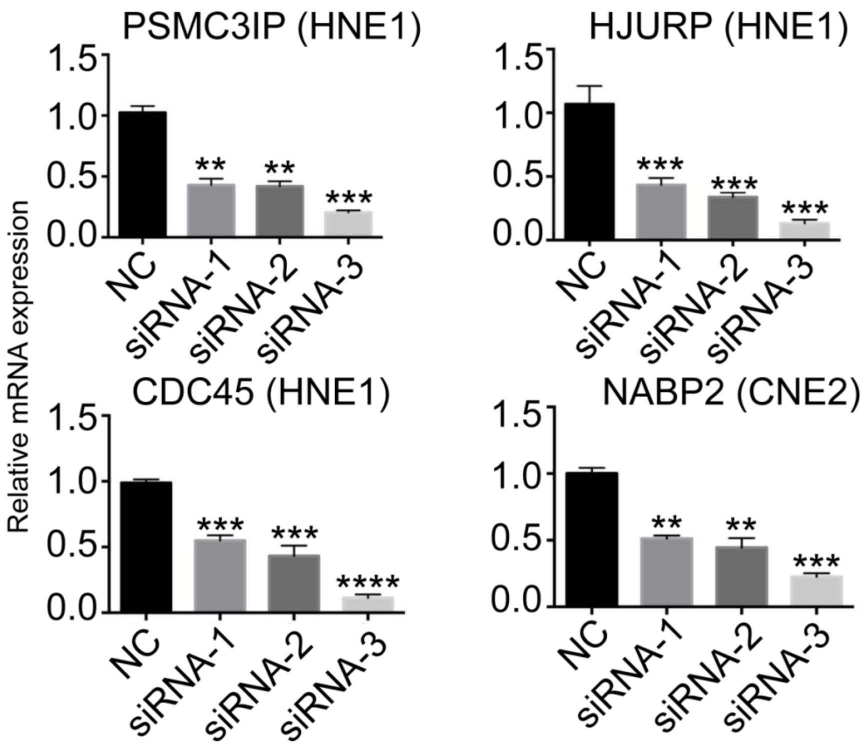

Figure S7. The expression levels of PSMC3IP, HJURP, CDC45 in HNE1 and NABP2 in CNE2 cells transduced with siRNA. ** represent P < 0.01, and *** represent P < 0.001.

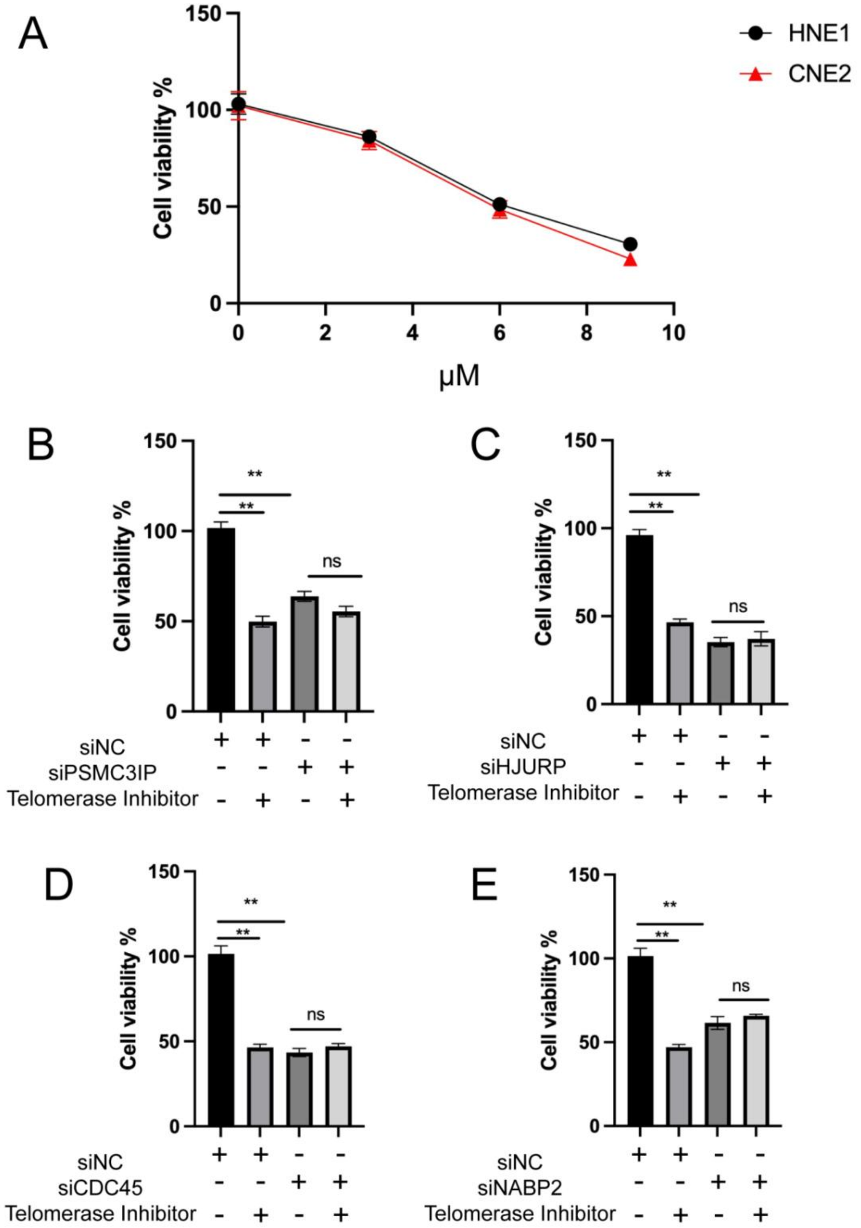

Figure S8. Effects of telomerase inhibitor on NPC cell proliferation. (A) Results of CCK-8 assay with different μM group for telomerase inhibitor. (B), (C), and (D) CCK-8 assays show that the effect of telomerase inhibitor in the cell proliferation for the inhibition of PSMC3IP, HJURP, CDC45 or not in HNE1 cells. (E) CCK-8 assays show that the effect of telomerase inhibitor in the cell proliferation for the inhibition of NABP2 or not in CNE2 cells. ** represent $P < 0.01$.

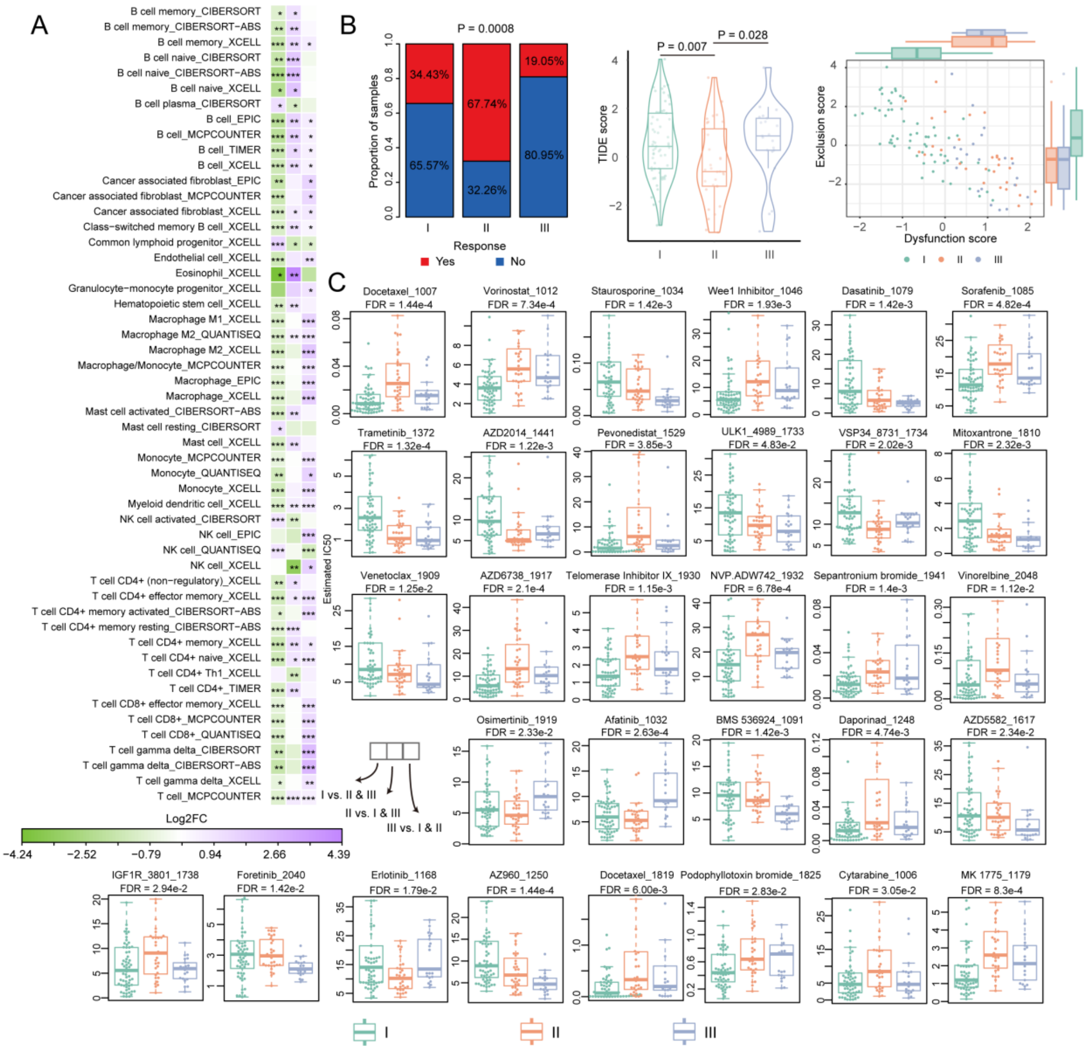

Figure S9. The TIME, immunotherapy response, and drug sensitive for original NPC clusters. (A) Heatmap showing the differences of immune cell infiltrations obtained from CIBERSORT, CIBERSORT-ABS, QUANISEQ, TIMER, EPIC, MCPCOUNTER, and xCell for original NPC clusters. (B) Bar plot showing the proportions of patients who responded to immunotherapy in original NPC subtypes. Box plot showing the differences of TIDE score between original NPC subtypes. The negative correlation between exclusion and dysfunction score for NPC patients. (C) The differences of estimated IC50 values for chemotherapeutics between original NPC subtypes. Only the IC50 values of drugs exhibited significantly varied between NPC clusters were shown. * represent FDR < 0.05, ** represent FDR < 0.01, *** represent FDR < 0.001.
